# Towards the Maturation and Characterization of Smooth Muscle Cells Derived from Human Embryonic Stem Cells

**DOI:** 10.1371/journal.pone.0017771

**Published:** 2011-03-10

**Authors:** Helena Vazão, Ricardo Pires das Neves, Mário Grãos, Lino Ferreira

**Affiliations:** 1 CNC - Center of Neurosciences and Cell Biology, University of Coimbra, Coimbra, Portugal; 2 Biocant - Center of Innovation in Biotechnology, Cantanhede, Portugal; Universidade de Sao Paulo, Brazil

## Abstract

In this study we demonstrate that CD34^+^ cells derived from human embryonic stem cells (hESCs) have higher smooth muscle cell (SMC) potential than CD34^−^ cells. We report that from all inductive signals tested, retinoic acid (RA) and platelet derived growth factor (PDGF_BB_) are the most effective agents in guiding the differentiation of CD34^+^ cells into smooth muscle progenitor cells (SMPCs) characterized by the expression of SMC genes and proteins, secretion of SMC-related cytokines, contraction in response to depolarization agents and vasoactive peptides and expression of SMC-related genes in a 3D environment. These cells are also characterized by a low organization of the contractile proteins and the contractility response is mediated by Ca^2+^, which involves the activation of Rho A/Rho kinase- and Ca^2+^/calmodulin (CaM)/myosin light chain kinase (MLCK)-dependent pathways. We further show that SMPCs obtained from the differentiation of CD34^+^ cells with RA, but not with PDGF_BB,_ can be maturated in medium supplemented with endothelin-1 showing at the end individualized contractile filaments. Overall the hESC-derived SMCs presented in this work might be an unlimited source of SMCs for tissue engineering and regenerative medicine.

## Introduction

Vascular smooth muscle cells (VSMCs) have enormous applications in regenerative medicine [1], [2], [3]. Studies have demonstrated that smooth muscle-like cells (SMLCs) can be derived from bone marrow-[4], [5], adipose-[6], [7] and umbilical cord blood-derived stem cells [8]. Due to the easy expansion, human embryonic stem cells (hESCs) represent an alternative source of VSMCs particularly for old patients having stem cells with impaired function. Recent studies reported different strategies to differentiate hESCs into SMLCs by exposing a monolayer of undifferentiated hESCs to retinoic acid [9] or a combination of cell culture medium and extracellular matrix environment [10], [11], [12] either in single-hESC- [13], embryoid bodies (EBs)- [12] or stromal cell- [14] culture conditions. In one case, SMLCs transplanted subcutaneously in an animal model were able to contribute for the formation of functional blood microvessels [12]. Despite these advances, several issues remain poorly understood: (i) what hESC-derived population has the most SMC potential, (ii) the bioactive molecules involved in the differentiation process, (iii) the modulatory effect of 3D environments in SMLCs, (iv) the functionality of the differentiated SMLCs, and (v) the level of organization of the contractile protein filaments.

Here we evaluate the smooth muscle cell (SMC) differentiation of different cell populations isolated from human embryoid bodies grown in suspension for 10 days. The isolated cells were cultured in media supplemented with several inductive signals, including platelet-derived growth factor (PDGF_BB_), retinoic acid (RA), transforming growth factor beta 1 (TGF_β-1_) or a combination of PDGF_BB_ with TGF_β-1_. We show that CD34^+^ cells have higher SMC potential than CD34^−^ cells and PDGF_BB_ and RA are the most effective agents to drive the differentiation of hESCs into smooth muscle progenitor cells (SMPCs). We further demonstrate that these cells contract and relax in response to SMC agonists or inhibitors, respectively, and the effect is mediated by Rho A/Rho kinase- and Ca^2+^/CaM/MLCK-dependent pathways. In addition, cells encapsulated in 3D gel scaffolds further differentiate towards SMC lineage as confirmed by gene analysis. Finally, we show that Endothelin-1 induces the organization of the contractile protein filaments.

## Materials and Methods

An expanded Materials and Methods section is provided in the online data supplement (Materials and Methods S1).

### hESC culture and embryoid body (EB) formation

Undifferentiated hESCs (passages 27–62; H9, WiCell, Wisconsin, http://www.wicell.org/) were grown on an inactivated mouse embryonic fibroblast (MEF) feeder layer, as previously described [12]. To induce the formation of EBs, the undifferentiated hESCs were treated with 2 mg/mL type IV collagenase (Invitrogen, http://www.invitrogen.com) for 2 h and then transferred (2∶1) to low attachment plates (Corning, http://www.corning.com) containing 10 mL of differentiation medium [80% KO-DMEM, 20% fetal bovine serum (FBS, Invitrogen), 0.5% L-glutamine, 0.2% β-mercaptoethanol, 1% nonessential amino acids and 50 U/ml∶50 µg/ml penicillin-streptomycin solution]. EBs were cultured for 10 days at 37°C, 5% CO_2_ in a humidified atmosphere, with media changes every 3–4 days.

### Isolation and differentiation of CD34^+^, CD34^−^ and CD34^+^KDR^−^ cells

CD34^+^ cells were isolated from EBs at day 10 according to a protocol previously reported by us [12]. For some experiments, the CD34^+^ cells were further separated in CD34^+^KDR^−^ cells. In this case, cells were labeled with anti-VEGF R2/KDR-PE antibody (R&D, http://www.rndsystems.com/), then conjugated with anti-PE antibody coupled with magnetic beads, and finally the magnetically labeled cells were separated into CD34^+^KDR^+^ and CD34^+^KDR^−^ using a MS-MACS column (Miltenyi Biotec, http://www.miltenyibiotec.com). Isolated cells were grown on 24-well plates (1.5×10^4^ cells/cm^2^) coated with 0.1% gelatin and containing one of the following media: smooth muscle growth medium-2 (SMGM-2), endothelial growth medium-2 (EGM-2) or EGM-2 supplemented with PDGF_BB_ (50 ng/mL, Prepotech, http://www.peprotech.com/) or RA (1 µM, Sigma, http://www.sigmaaldrich.com) or TGF_β-1_ (10 ng/mL, Prepotech) or a mixture of PDGF_BB_ with TGF_β-1_ (50 ng/mL; 10 ng/mL). Human vascular smooth muscle cells (hVSMCs, isolated from the arteries of human umbilical cord, Lonza, http://www.lonza.com) were used as controls for the differentiation studies. Cells were cultured in SMGM-2 media (Lonza; until passage 6) being the medium changed every 2 days.

### Immunofluorescence analysis

Cells were transferred to gelatin-coated slides containing differentiation medium, allowed to attach overnight, and then fixed with 4% paraformaldehyde (Electron Microscopy Sciences, http://www.emsdiasum.com/microscopy) for 15 min at room temperature. Cells were blocked with 1% (w/v) BSA and stained for 1 h with anti-human primary antibodies specific for smooth muscle α-actin (α-SMA, 1A4, Dako, http://www.dako.com/), smooth muscle myosin heavy chain (SM-MHC, SMMS-1, Dako) and calponin (CALP, Calponin1, Santa Cruz Biotec, http://www.scbt.com/). In each immunofluorescence experiment, an isotype-matched IgG control was used. Binding of primary antibodies to specific cells was detected with anti-mouse IgG Cy3 conjugate or anti-mouse IgG FITC (both form Sigma). Cell nuclei were stained with 4′, 6′-diamidino-2-phenylindole (DAPI) (Sigma) and the slides examined with either a Zeiss fluorescence microscope or Zeiss LSM 50 confocal microscope.

### Reverse transcription-polymerase chain reaction (RT-PCR) analysis

Total RNA from experimental groups was isolated using a protocol with TRIzol (Invitrogen) and RNeasy Minikit (Qiagen, Valencia, http://www1.qiagen.com/). cDNA was prepared from 1 µg total RNA using Taqman Reverse transcription reagents (Applied Biosystems, CA). Quantitative PCR (qPCR) was performed using Power SYBR Green PCR Master Mix and the detection using an ABI PRISM 7500 System (Applied Biosystems, http://www3.appliedbiosystems.com). Quantification of target genes was performed relative to the reference GAPDH gene: relative expression = 2^[−(Ct^_sample_^−Ct^_GADPH_^)]^. The mean minimal cycle threshold values (Ct) were calculated from quadruplicate reactions. Then, the relative gene expression in each experimental group was normalized to the relative gene expression found in hVSMCs. Primer sequences are published as supporting information (**Table S1**). For the RT^2^ Profiler™ PCR Array, cDNA was prepared from 1 µg total RNA using the RT^2^ PCR Array first strand kit (SABiosciences, http://www.sabiosciences.com/). RT-PCR assays were performed using the human extracellular matrix and adhesion molecules RT^2^ Profiler™ PCR Array (SABiosciences) on an ABI PRISM 7500 System. Data analysis was performed using analysis software provided by the kit manufacturer.

### Intracellular Ca^2+^ variation measurements

SMCs or hESC-derived cells were loaded with Fura-2 calcium fluorescent indicator [15] by incubation with 5 µM of the membrane permeable acetoxymethyl (AM) derivative FURA-2/AM (1 mM in DMSO, Molecular Probes, Invitrogen) and 0.06% (w/v) Pluronic F-127 (Sigma), using basal medium (M199, Sigma) as a vehicle (35 µl/well, not supplemented with serum nor antibiotics), for 1 h at 37°C in 5% CO_2_ and 90% humidity. Cells were then stimulated with 100 µM histamine (Sigma) [16], 10^−7^ M bradykinin (Calbiochem, http://www.merck-chemicals.com/) [17], 10^−5^ M angiotensin II (Calbiochem) [17], 10^−5^ M carbachol (AlfaAesar) [6] or 50 mM KCl (Merck, http://www.merck-chemicals.com) [16] by adding 1 µl of a stock solution. A detailed methodology for the fluorescence acquisition can be found on the online data supplement (Materials and Methods S1).

### Contractility assays

Agonist-induced contractile activity of the differentiated cells was evaluated as previously described [6], [12]. hECS-derived cells cultured for 3 passages were washed with DMEM and contraction was induced by incubating these cells with 10^−5^ M carbachol in DMEM (Sigma) medium for 30 min. Contraction was calculated as the difference in cell area between time zero and 30 minutes. The same microscopic fields were imaged before and after treatment for contraction analysis. In a distinct experiment, cell relaxation was induced by incubation with 10^−4^ M atropine (AlfaAesar) in DMEM for 1 h followed by contraction with 10^−5^ M carbachol. Contraction was calculated as before. hVSMCs (3rd passage) were used as controls.

### Effect of RhoA/Rho kinase- and CaM/MLCK- agonists in cell contraction and maturation

hVSMCs or hESC-derived SMPCs were treated with the inhibitor W-7 (12 µg/mL, Sigma) for 30 min and the agonist U46619 (1 µM, a CAM kinase-agonist) (Cayman Chemicals, http://www.caymanchem.com) in serum-free M199 medium for 3 days. At the end, cells were characterized for the expression of SMC markers by immunostaining. Finally, the contraction of U46619-treated cells was evaluated by embedding the cells in fibrin gels (3.5×10^4^ cells/50 µL) and measuring gel size at time 0 and 14 h by microscopy. This methodology was repeated to evaluate the effect of Rho kinase-agonist in cell contraction and maturation. In this case, the cells were exposed to the inhibitor Y27632 (13 µM) (Cayman Chemicals) and the agonist endothelin-1 (End-1, 10 nM, Sigma).

### Cytokine measurements

Cell culture supernatants were assayed for cytokines using a Bio-plex human 17-plex panel immunoassay kit (Bio-Rad, http://www.bio-rad.com) and cytokines concentrations were determined using Bio-Plex Manager 5, according to manufacturer's instructions. Samples and controls were run in triplicate, standards and blanks in duplicate. A detailed methodology can be found on the online data supplement (**Materials and Methods S1**).

### Co-culture of hESC-derived SMPCs with hVSMCs

To evaluate the effect of both soluble and insoluble factors, fluorescently-labeled hESC-derived SMPCs (stained with PKH67 dye, Sigma) were plated on top of mytomycin-inactivated hVSMC cultured on a 24 well plate for 2 days before use. This co-culture system was maintained for 5 days after which the overall cells were tripsinized, and fluorescent (PKH67^+^ cells) cells sorted by a FACS Aria (BD). The sorted cells were characterized by immunohistochemistry to evaluate the expression and organization of α-SMA, SM-MHC and calponin filaments.

### Culture of SMPCs in three-dimensional gels

Fibrin gels were obtained by the crosslinking of 20 mg/mL fibrinogen/TBS pH 7.4 in the presence of 50 U/mL thrombin/TBS pH 7.4 (both from Sigma-Aldrich). Fibrin gels (50 µL) were prepared by mixing the following components: 10 mg/mL fibrinogen, 2.5 mM CaCl_2_, 2 U/mL thrombin and 0.01 mg/mL aprotinin (Sigma-Aldrich). This solution was allowed to gel at 37°C in 100% relative humidity. hVSMC or hESC-derived SMPCs (3×10^5^) were encapsulated in fibrin gels (50 µl). Cells were centrifuged and resuspended in the fibrin gel precursor solution and included in 1 mL sterile syringes with cut tips. Polymerization was initiated at 37°C and allowed to proceed over 30 min. After polymerization, the cell constructs were removed from the syringe and placed in 24-well plates, containing specific medium, for up to 96 h.

### Statistical analysis

An unpaired *t* test or one-way ANOVA analysis of variance with Bonferroni post test was performed for statistical tests using SigmaStat. Results were considered significant when *P*<0.05. Data are shown as mean ± SEM.

## Results

### Effect of initial cell population and inductive signals on cell proliferation

**Figure 1:**
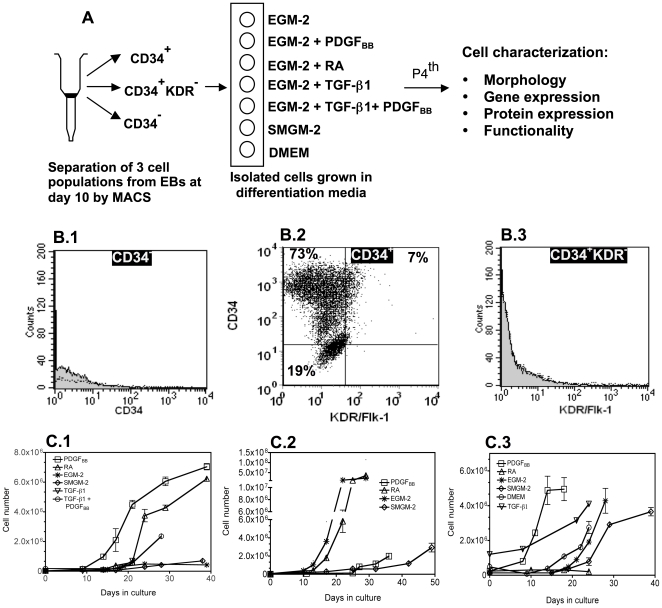
The effect of initial cell population and inductive signals on cell proliferation. (A) Protocols adopted to drive the differentiation of CD34^+^, CD34^+^KDR^−^ and CD34^−^ cells isolated from EBs at day 10 into the SMC lineage. (B) Flow cytometric analysis of hES-derived cells: CD34^−^ (B.1), CD34^+^ (B.2) and CD34^+^KDR^−^ (B.3) cells (in this last case isolated from the CD34^+^ cell population). The results show that CD34^−^ cells do not express CD34^+^ marker (B.1), CD34^+^ cells are formed by CD34^+^KDR^−^ and CD34^+^KDR^+^ cells (B.2), and CD34^+^KDR^−^ do not express the KDR marker (B.3). Percent of positive cells (dash plot) were calculated based in the isotype controls (grey plot). (C) Time-course proliferation of CD34^+^ (C.1), CD34^+^KDR^−^ (C.2) and CD34^−^ cells (C.3).

We evaluated the SMC differentiation potential of three cell populations isolated from EBs at day 10: CD34^+^ cells, CD34^−^ cells and CD34^+^KDR^−^ cells (mesenchymal origin [18]) (**Figure 1A**). The percentages of CD34^+^ and CD34^+^KDR^−^ cells in EBs were approximately 2% and 1.7%, respectively, after MACS isolation. As evaluated by flow cytometry (**Materials and Methods S1**), the purity of the cell populations CD34^+^, CD34^−^, and CD34^+^KDR^−^ was >80%, >98% and >98%, respectively (**Figure 1B**). These cells were plated on gelatin coated dishes, at low density (1.5×10^4^/cm^2^), and cultured on basal media (EGM-2 or SMGM-2) supplemented or not with different inductive signals to guide their SMC differentiation: PDGF_BB_ (50 ng/mL) [5], [12], [19], RA (1 µM) [9], [17], [20], TGF_β-1_ (10 ng/mL) [5], [21], [22] or a combination of TGF_β-1_ (10 ng/mL) with PDGF_BB_ (50 ng/mL) (**Figure 1A**). These concentrations have been used previously in the differentiation of stem cells from different origins into vascular cells [5], [9], [21].

CD34^+^ cells cultured on EGM-2 medium supplemented with PDGF_BB_ presented the highest proliferation (more than 8 population doublings over 20 days), followed by the ones cultured in EGM-2 medium supplemented with RA (**Figure 1C.1**). CD34^+^ cells grown in EGM-2 medium without PDGF_BB_ or RA proliferated poorly over 40 days (**Figure 1C.1**). Interestingly, CD34^+^ cells cultured in medium supplemented with TGF_β-1_ and PDGF_BB_ had low proliferation suggesting that TGF_β-1_ inhibited the effect of PDGF_BB_. CD34^+^KDR^−^ cells cultured on EGM-2 medium without any supplements proliferated extensively, showing more than 8 population doublings over 20 days (**Figure 1C.2**). Similar proliferation potential was observed for CD34^+^KDR^−^ cells grown in EGM-2 medium supplemented with RA, but not on medium supplemented with PDGF_BB_.

The proliferation rate of CD34^−^ cells was also assessed in the media formulations tested for CD34^+^ and CD34^+^KDR^−^ cells. CD34^−^ cells cultured on EGM-2 medium supplemented with PDGF_BB_ showed the highest proliferation, having more than 8 population doublings over an 18 days period (**Figure 1C.3**). In contrast, cells grown in EGM-2 medium supplemented with RA did show a poor proliferation over more than 20 days.

All taken together, the proliferation potential of the cells was dependent on the initial cell population and the supplements added to the basal media.

### Effect of initial cell population and inductive signals on SMC differentiation

**Figure 2:**
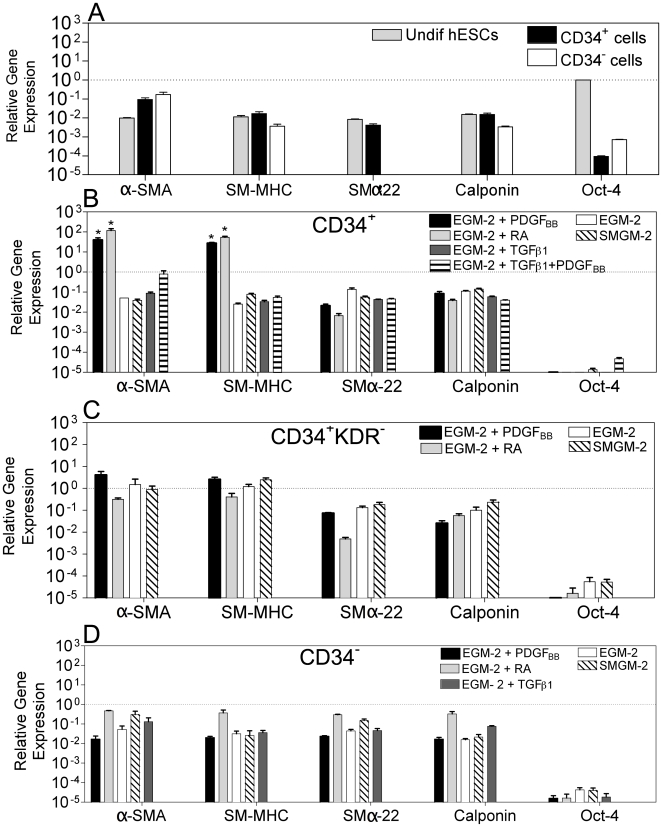
Gene expression in hESC-derived cells evaluated by qRT-PCR. Gene expression in each experimental group was normalized by the corresponding gene expression observed in hVSMCs. Oct-4 was normalized by the expression in undifferentiated hESCs. (A) Gene expression of hESCs, CD34^+^ and CD34^−^ cells before differentiation. SMα-22 expression in CD34^−^ cells is very low (<8.5×10^−6^) and not visible in the graph. CD34^+^ (B), CD34^+^KDR^−^ (C), and CD34^−^ (D) cells were cultured in SMGM-2 medium, EGM-2 medium, and EGM-2 medium supplemented with PDGF_BB_ or TGF_β-1_ or RA or TGF_β-1_ plus PDGF_BB_. Cells were characterized at passage 4 (≈20 days). Results are Mean ± SEM (*n* = 4); * denotes statistical significance (*P*<0.05).

Next we evaluated the expression of SMC-specific genes in the hESC-derived cells (passage 4) by quantitative RT-PCR (**Figure 2**). The genes analyzed included: α-smooth muscle actin (α-SMA), an early marker of SMC differentiation and highly specific marker for SMCs in adult animals [23]; smooth muscle myosin heavy chain (SM-MHC), a later marker in SMC differentiation that seems to be highly restricted to SMCs [24]; calponin and smooth muscle α-22, definitive SMC markers [25]. The gene expression in the hESC-derived cells was normalized by the corresponding gene expression in hVSMCs.

Undifferentiated CD34^+^ cells expressed low levels of SM-MHC (∼2.0%), SMα-22 (<1%) and calponin (∼2.0%), in most cases comparable to the levels found in undifferentiated hESCs, and moderate levels of α-SMA (∼10%) (**Figure 2**). Culture of these cells in the presence of EGM-2 medium supplemented with PDGF_BB_ or RA contributed for the up-regulation of SMC genes, as confirmed by the significant increase in the expression of α-SMA (>400-fold) and SM-MHC (>1,600-fold) when compared with undifferentiated CD34^+^ cells (p<0,05), and hVSMCS (α-SMA: >39-fold; SM-MHC: >27-fold) (**Figure 2**). Addition of TGF_β-1_, or TGF_β-1_ plus PDGF_BB_ increased the cellular expression of α-SMA, SM-MHC, SMα-22 and calponin when compared to undifferentiated CD34^+^ cells; however, the expression was one order of magnitude lower than the one in hVSMCS, suggesting that the CD34^+^-derived cells were not fully differentiated into SMCs.

CD34^+^KDR^−^ cells cultured in SMGM-2 medium or EGM-2 medium in the presence or absence of PDGF_BB_ expressed higher levels of α-SMA (>42-fold), SM-MHC (>150-fold), SMα-22 (>18-fold) and calponin (>2-fold) than undifferentiated CD34^+^ cells (**Figure 2**). In addition, these cells showed similar levels of expression of α-SMA and SM-MHC to the one found in hVSMCs, suggesting that these cells shared features with SMCs.

In contrast to the CD34^+^ and CD34^+^KDR^−^ cells, CD34^−^ cells cultured in several media formulations had lower expression of SMC markers, indicating low efficiency of differentiation into SMCs (**Figure 2**). Of note, all the hESC-derived cells previously described had low expression of Oct-4 confirming their loss of pluripotency. In addition, hESC-derived cells expressing high levels of SMC markers had no expression of PECAM-1 (endothelial cell marker) and α-actinin (a marker of cardiomyocytes) (data not shown) confirming again their SMC differentiation.

Next, the expression and filament organization of contractile proteins was evaluated in cell populations having similar or higher α-SMA and SM-MHC gene expression than hVSMCs, i.e., CD34^+^RA, CD34^+^PDGF_BB_, CD34^+^KDR^−^PDGF_BB_ and CD34^+^KDR^−^EGM-2. All hESC-derived cells stained positive for α-SMA, SM-MHC and calponin (**Figure S1 and Figure S2**). More than 70% of the cells expressed α-SMA as evaluated by FACS analyses (**Figure S3**). Individualized calponin filaments were observed in 16 to 60% of the overall cells; however, organized α-SMA protein filaments were only observed in CD34^+^PDGF_BB_ (6.0%) and CD34^+^KDR^−^EGM-2 (6.1%) cells, and no organized SM-MHC filaments were observed in the hESC-derived populations (**Figure S3**). In contrast, hVSMCs expressed high levels of organized α-SMA (84.6%), SM-MHC (94.6%) and calponin (80.1%) filaments.

Collectively, gene and protein analyses indicate that several inductive signals (PDGF_BB_, or RA or EGM-2 basal medium) are able to drive the SMC differentiation of CD34^+^ or CD34^+^KDR^−^ cells, characterized by variable expression of SMC proteins and minimal assembly of the α-SMA protein into filaments.

### Some hESC-derived cells present a secretion profile similar to hVSMCs

**Figure 3:**
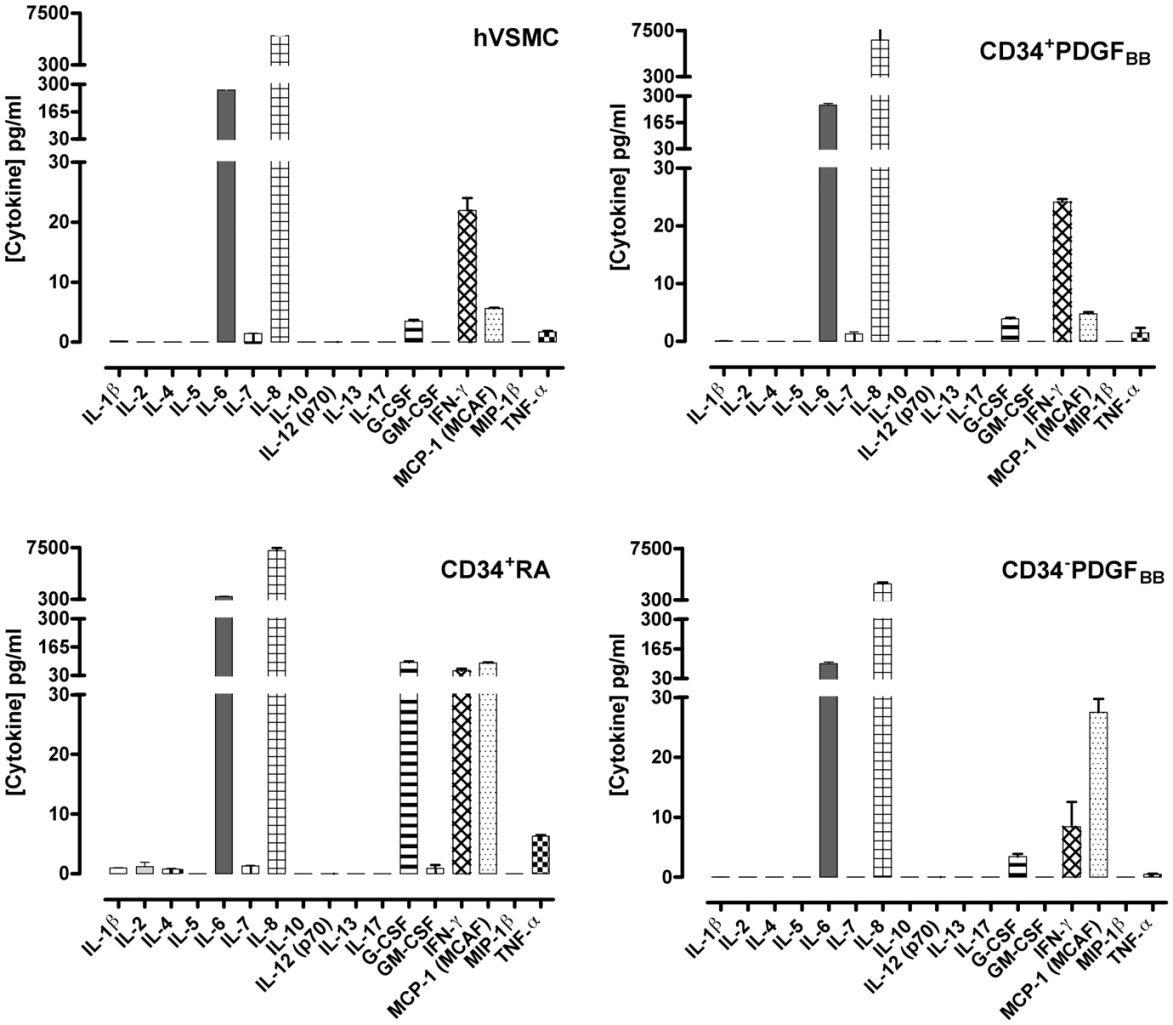
Secretomic profile of hVSMC and hECS-derived cells. Seventeen cytokines were measured simultaneously in medium collected from hVSMC, CD34^+^PDGF_BB_, CD34^+^RA, and CD34^−^PDGF_BB_ cells at passage 4. Results are Mean ± SEM (*n* = 3).

Using the multiplex cytometric bead array method we investigated the secretion of 17 analytes by hVSMC, CD34^+^PDGF_BB_, CD34^+^RA, CD34^+^KDR^−^PDGF_BB_, CD34^+^KDR^−^EGM-2 and CD34^−^PDGF_BB_ cells. Out of the 17 analytes analyzed, hVSMCs secreted IL-6, IL-7, IL-8, G-CSF, IFN-γ, MCP-1 (MCAF) and TNF-α, above the detection limit (>0.2 pg/mL) (**Figure 3**). Cytokines IL-6 and IL-8 were the most secreted cytokines. CD34^+^PDGF_BB_ and CD34^+^KDR^−^EGM-2 cells secreted the same analytes as hVSMCs. CD34^+^KDR^−^PDGF_BB_ and CD34^−^PDGF_BB_ cells did not secrete IL-7, while CD34^+^RA cells secreted all seven cytokines mentioned and 4 cytokines more (IL-1β, IL-2, IL-4 and GM-CSF) (**Figure S4**).

Interestingly, the secretion profile of CD34^+^PDGF_BB_ cells is very similar to the one observed for hVSMCS, either in the type and concentration of the analytes secreted (**Figure 3**). In contrast, CD34^−^PDGF_BB_ cells secreted analytes at different concentration than hVSMCs, and the other cell populations seemed to be in an intermediate stage in terms of secretion profile (**Figure 3** **and Figure S4**). CD34^+^RA cells secreted higher levels of cytokines than all the remaining cell populations, including hVSMCs.

### Some hESC-derived cells like hVSMCs have the ability to contract when treated with vasoactive agonists and the process is mediated by Ca^2+^

**Figure 4:**
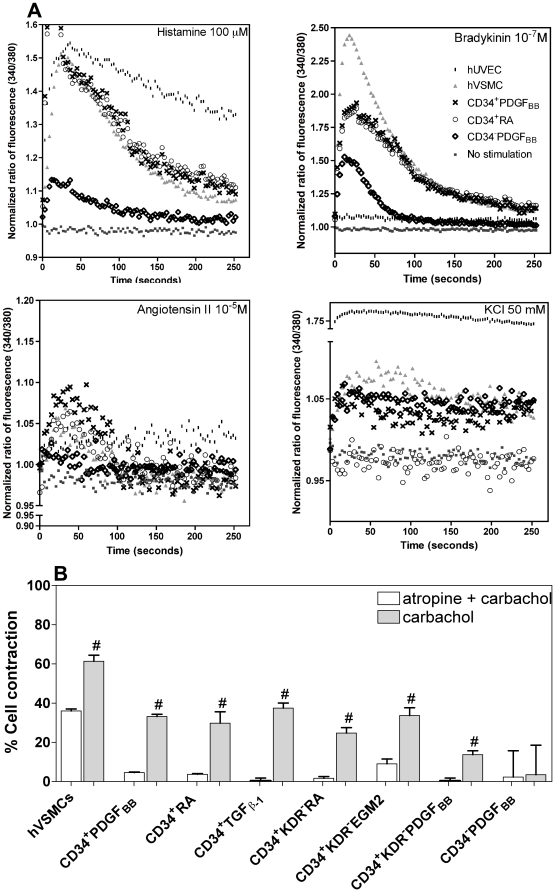
Contractility of hESC-derived cells. (A) Concentration of intracellular Ca^2+^ in FURA-2-loaded cultured hESC-derived cells in response to several agonists (100 µM histamine, 10^−7^ M bradykinin, 10^−5^ M angiotensin II or 50 mM KCl). hVSMCs and HUVECs were used as positive and negative controls, respectively. Traces are representative of 4 independent experiments for each condition. (B) Contractility of hESC-derived cells when exposed to the effects of carbachol (10^−5^ M in DMEM medium, for 30 min) or atropine (10^−4^ M, 1 h) plus carbachol (10^−5^ M for 30 min). hVSMCs were used as controls. Results are Mean ± SEM (*n* = 3); ^#^ denotes statistical significance (*P*<0.05) in the same experimental group.

The ability of SMCs to contract in response to vasoactive agonists is mediated by an increase of intracellular Ca^2+^ levels which triggers the SMC contractile apparatus [26]. To evaluate whether hESC-derived cells had contractility mediated by Ca^2+^, the cells were loaded with FURA-2, a Ca^2+^ sensitive dye, and their response to vasoactive agonists (bradykinin, angiotensin II, carbachol and histamine) and depolarization agents (KCl) was monitored by fluorescence (**Figure 4A**). The response profile was compared to the one observed for hVSMCs and human umbilical vein endothelial cells (HUVECs), as positive and negative controls, respectively. Intracellular free Ca^2+^ [Ca^2+^]_i_ levels increase when hVSMCs are exposed to bradykinin, angiotensin II and carbachol, while no measurable effect is observed in HUVECs. KCl induces a higher increase in the [Ca^2+^]_i_ levels in HUVECs than in hVSMCs, while histamine induces similar levels of [Ca^2+^]_i_ in both cell types but following different profiles. These response patterns are typical for HUVECs and hVSMCs [27], [28], [29], [30], [31].

CD34^+^RA and CD34^+^PDGF_BB_ cells exposed to histamine, bradykinin, angiotensin II, carbachol or KCl had similar response profiles as observed for hVSMCs (**Figure 4A** **and Figure S5**). For bradykinin and KCl the response intensity was lower than in hVSMCs, however similar intensity was observed for angiotensin II, carbachol and histamine. On the other hand, although CD34^+^KDR^−^RA, CD34^+^KDR^−^PDGF_BB_ and CD34^+^KDR^−^EGM-2 cells had similar response profiles as hVSMCs, in general the intensity of response was lower (**Figure S6**). In contrast to the previous hESC-derived populations, CD34^−^PDGF_BB_ cells showed a very low variation in the intracellular levels of Ca^2+^ when exposed to depolarization and vasoactive peptides.

To examine whether hESC-derived cells were able to contract, they were subjected to the effects of carbachol and atropine (**Figure 4B**). With the exception of CD34^−^PDGF_BB_ cells, all cells were able to contract after exposure to carbachol (13 to 38% contraction after 30 min, depending on the cell population). In most cases, cell contraction was not significantly different (*P*>0.05) from the one observed for hVSMCs. Moreover, with the exception of CD34^−^PDGF_BB_ cells, the muscarinic inhibitor atropin significantly blocked or reduced the carbachol-mediated effect (*P*<0.05) (**Figure 4B**).

Based on the expression of SMC proteins and genes, secretion of cytokines and cellular contraction, CD34^+^PDGF_BB_ and CD34^+^RA cells were selected for further characterization. These cells are likely at a smooth muscle progenitor cell stage (SMPCs), which can potentially be further induced into a more mature SMC phenotype.

### The contraction of both hESC-derived SMPCs and hVSMCs involves Rho A/Rho kinase- and Ca^2+^/CaM/MLCK-dependent pathways

**Figure 5:**
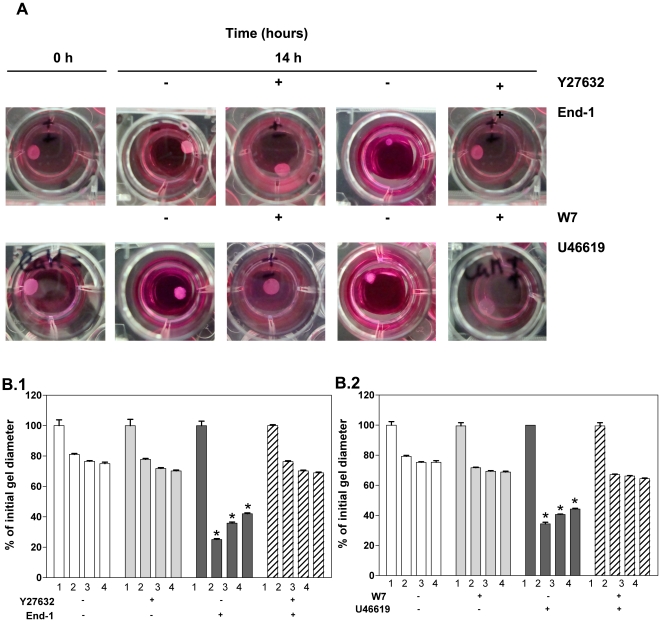
Evaluation of the role of Rho A/Rho kinase- and Ca^2+^/CaM/MLCK-dependent pathways in the contraction of SMPCs and hVSMCs. CD34^+^PDGF or CD34^+^RA cells were initially treated for 3 days with serum-free medium containing the agonists endothelin-1 (End-1 (10 nM), A and B.1) or U46619 (1 µM) (A and B.2) in the presence or absence of the Rho-kinase inhibitor Y27632 (13 µM) or the calmodulin inhibitor W-7 (12 µg/mL). The cells were then encapsulated in fibrin gels and the diameter of the gel assessed at time 0 (1) and 14 h (2,3,4). Graph displays the variation in gel diameter (percentage) encapsulating hVSMCs (2), CD34^+^PDGF_BB_ cells (3) or CD34^+^RA cells (4). Data are shown as mean ± SEM (*n* = 6), * *P*<0.001 relatively to samples inhibited with Y27632 or W-7, by Student's *t*-test.

CaM/MLCK- and RhoA/Rho kinase-dependent pathways play a pivotal role in SMC contraction [7], [26]. The Ca^2+^/CaM pathway plays a key role in SMC contraction through the stimulation of MLCK-mediated phosphorylation of myosin light chain 20,000 Da (MLC_20_) [26]. To assess whether Ca^2+^/CaM pathway was involved in the contraction of hESC-derived SMPCs, cells were exposed to the CaM-specific inhibitor W-7 [32], and then contraction was induced by exposing them to the CaM agonist U46619 [7]. To facilitate the evaluation of cell contraction, cells were encapsulated in fibrin gels, and gel diameter was determined after 14 h. Pre-incubation of hESC-derived SMPCs with W-7 significantly inhibited U46619-induced contraction (**Figure 5A** **and** **Figure 5B**). Similar results were obtained for the control cells hVSMCs. Overall the results indicate the involvement of CaM/MLCK-kinase pathway in cell contraction.

To examine whether Rho kinase was involved in the hESC-derived SMPC contraction, the cells were pre-treated with the Rho kinase-specific inhibitor Y27632 [7] and then contraction was induced by the agonist End-1 [33]. Cells treated with Y27632 and End-1 showed no significant contraction (**Figure 5A** **and** **Figure 5B**). In contrast, cells treated with End-1 but not Y27632 contracted significantly. Similar response profiles were obtained for hVSMCs and hESC-derived SMPCs. Collectively the results indicate the involvement of a Rho/Rho kinase-dependent pathway in SMC contraction.

### 3D culture of hESC-derived SMPCs modulates gene expression towards the expression observed in hVSMCs

We investigated the use of hESC-derived SMPCs for tissue engineering applications [2], [3]. Fibrin gels previously used for the encapsulation of neonatal SMCs [34] were selected as scaffolds for the encapsulation of SMPCs. These gels allow cell attachment and can be remodeled by cellular metalloproteinases. SMPCs were encapsulated in fibrin gels for 3 days after which the cells were characterized at protein and gene levels.

**Figure 6:**
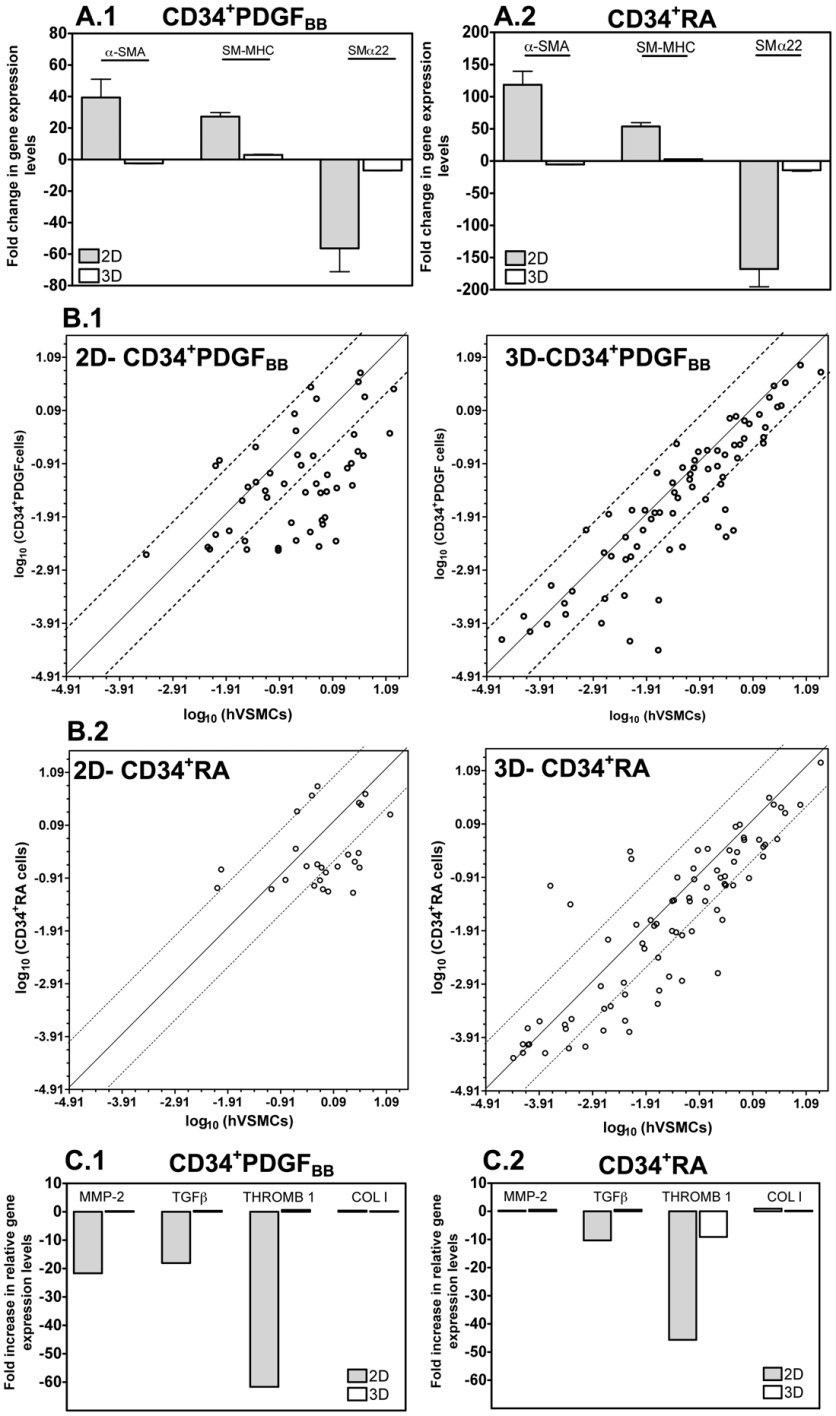
Characterization of hESC-derived SMPCs encapsulated in 3D fibrin gel scaffolds. SMPCs were characterized after being encapsulated in fibrin gels for 3 days. (A) SMC gene expression on CD34^+^RA (A.1) and CD34^+^PDGF (A.2) cells, as assessed by qRT-PCR. Gene expression of hESC-derived SMPCs was normalized by gene expression of hVSMCs under the same culture conditions. Results are Mean ± SEM (*n* = 4). (B) Comparison of extracellular matrix and adhesion molecules-related gene expression in CD34^+^PDGF_BB_ (B.1) or CD34^+^RA cells (B.2) with hVSMCs cultured in 2D (tissue culture polystyrene) or cultured in 3D fibrin gels. Gene expression was evaluated using a RT^2^ Profiler™ PCR array. (C) Normalized extracellular matrix and soluble factor gene expression of SMPCs relatively to hVSMCs, both cultured in 3D or 2D systems.

Gene expression of SMPCs (CD34^+^RA and CD34^+^PDGF) was compared to hVSMCs under the same culture conditions (**Figure 6A** **and** **Figure 6B**). The culture of SMPCs in 3D gels modulated the expression of SMC genes (α-SMA, SM-MHC or SMα-22) towards the one observed for hVSMCs cultured in 3D gels. We complemented these studies by evaluating the expression of extracellular matrix and adhesion molecules by a quantitative real-time PCR array. This array evaluated the expression of 84 genes involved in cell-cell and cell-matrix interactions. Again, the 3D culture of SMPCs modulated extracellular matrix and adhesion molecule genes towards the expression observed in hVSMCs (**Figure 6B**). The number of genes with similar expression increased from 23 to 58 or 9 to 53 when CD34^+^PDGF_BB_ cells or CD34^+^RA cells are cultured in 2D or 3D, respectively. Finally, gene expression associated with SMCs including collagen I and thrombospondin 1 [35], integrins α2, α3, α5, αV and β1 [36], the enzyme metalloproteinase 2 [37], and the growth factor TGF_β-1_ was similar in SMPCs and hVSMCs (**Figure 6C** **and Figure S7**).

### Co-culture of hESC-derived SMPCs with hVSMCs induces the assembly of α-SMA and calponin into filaments

**Figure 7:**
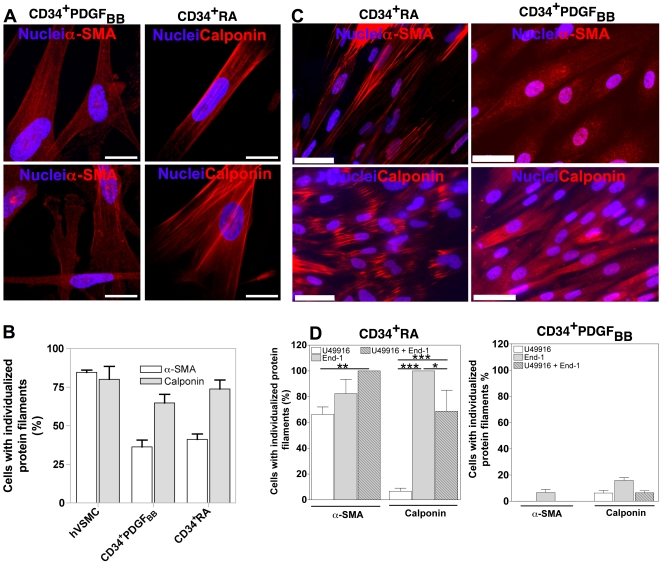
Maturation of hESC-derived SMPCs. (A and B) Expression and organization of SMC proteins on hESC-derived SMPCs cultured on top of inactivated-hVSMCs for 5 days. hVSMCs were used as controls. Bar corresponds to 50 µm. Results are Mean ± SEM (*n* = 10). (C and D) Expression and organization of SMC proteins on hESC-derived SMPCs treated with vasoactive agents for 3 days. Bar corresponds to 50 µm. Results are Mean ± SEM (*n* = 8); *, **, *** denote statistical significance (*P*<0.05, *P*<0.01, *P*<0.001, respectively).

Next we sought to investigate whether hESC-derived SMPCs could be further matured into a SMC phenotype with an organized contractile filament network. To accomplish this goal, hESC-derived SMPCs were initially labeled with PKH67 fluorescent dye and co-cultured on top of hVSMCs for 5 days. After this time, the cells were sorted and characterized by immunocytochemistry. Remarkably, hESC-derived SMPCs showed significant improvement in the organization of the fibers after contact with hVSMCs: 36.3% and 41.2% of CD34^+^PDGF_BB_ and CD34^+^RA cells, respectively, had organized α-SMA fibers, while 64.8% and 73.8% had organized calponin fibers (**Figure 7A** **and** **Figure 7B**). These results show that hESC-derived SMPCs are plastic cells and can be induced to differentiate into a more mature SMC phenotype displaying an organized contractile network.

### Vasoactive agents U46619 and End-1 improve the organization of contractile protein fibers

Next, we identified molecules able to maturate the hESC-derived SMPCs into SMCs having an organized contractile network. We sought to evaluate the effect of the agonists U46619 and End-1 involved in the CaM/MLCK- and RhoA/Rho kinase-contraction pathways, respectively. In CD34^+^RA cells, each agonist independently improved dramatically the organization of α-SMA and calponin fibers, being End-1 the most effective. The addition of both agonists did not significantly improve the effect of End-1 (**Figure 7C** **and** **Figure 7D**). Importantly, the induction effect of the vasoactive agents was not observed on CD34^+^PDGF_BB_ (**Figure 7D**) or CD34^−^PDGF_BB_ cells (**Figure S8**) showing that the inductive effect is dependent on the differentiation history of the SMPCs. The mature SMCs (derived from CD34^+^RA) express SMC markers including α-SMA, SM-MHC and calponin but not the cardiac marker α-actinin (**Figure S9**). Furthermore, the cells show no expression of troponin T, a protein found in skeletal and cardiac muscle but not in smooth muscle and vascular endothelial-cadherin, a protein typically expressed in endothelial cells (**Figure S10**).

## Discussion

In this study, we demonstrate that CD34^+^ cells have higher SMC potential than CD34^−^ cells. We further show that RA or PDGF_BB_ drive the differentiation of CD34^+^ cells into SMPCs We have characterized the differentiated cells at gene and at protein level, their secretome, the ability to contract when incubated with several pharmacological agents, and contraction mechanism mediated by Ca^2+^. Envisioning the use of these cells for vascular engineering, they were encapsulated on 3D fibrin scaffolds and characterized at gene and functional levels. Finally, we identified End-1 as a key molecule to induce the polymerization of contractile proteins in SMPCs (CD34^+^RA cells).

We examined the capacity of three hESC populations (CD34^+^, CD34^+^KDR^−^, CD34^−^) isolated from EBs at day 10, and cultured as single cells on media supplemented with inductive signals including PDGF_BB,_ RA, TGF_β-1_ and TGF_β-1_ plus PDGF_BB_ to differentiate into SMCs. Notably, TGF_β-1_ [5], [21], [38], PDGF_BB_ [5], [12], [19], and RA [9], [17] have been reported to be very important inductive signals for SMC differentiation from different initial stem cell populations. We report that from all inductive signals tested in this study, RA and PDGF_BB_ are the most effective in guiding the differentiation of hESC-derived CD34^+^ cells into SMPCs, based on gene and protein analysis, response to the depolarization agents and vasoactive peptides, contraction profile, secretion of cytokines, and behavior in a 3D scaffold. Although SMPCs express most of the SMC markers, they exhibit disorganized contractile proteins.

We further show that CD34^+^ cells have higher propensity to yield contractile SMPCs than CD34^−^ cells, when exposed to the same inductive signals. Interestingly, CD34^+^KDR^−^ cells which have been reported to be of mesenchymal origin [18], can give rise to SMPCs but they respond less efficiently to vasoactive peptides and depolarization agents, have lower contractile properties than CD34^+^PDGF cells, and have a different cytokine secretion profile as compared to hVSMCs. In line with previous results [39], our results suggest that cells expressing KDR receptor are needed for an efficient SMC differentiation.

The development of mature contractile SMCs from stem cells occurs in multiple steps comprising (i) the commitment to the SMC lineage, (ii) the differentiation into early immature and (iii) the maturation into the mature contractile phenotype [40]. Previously, we have reported that CD34^+^ cells could give rise to SMLCs when cultured in medium supplemented with PDGF_BB_; however, the differentiation of SMLCs was not complete since the assembly of α-SMA or SM-MHC proteins into filaments was not observed [12]. Curiously, similar results have been obtained in this work for the cell populations tested and exposed to different inductive signals including RA and TGF_β-1_.

Our results indicate that the co-culture of SMPCs with fully differentiated hVSMCs induces the assembly of α-SMA and calponin proteins into individualized filaments. This indicates that the cells are able to maturate into a fully contractile phenotype. It is known that both assembly and disassembly of actin filaments is regulated by RhoA [41]. Consistent with this, our results show that End-1, an agonist of RhoA pathway, dramatically increases actin polymerization in CD34^+^RA cells and the cells exhibit individualized α-SMA and calponin filaments. However, such inductive effect was not observed in CD34^+^PDGF_BB_ cells, and this could be due to the inhibition of mature SMC marker expression by PDGF_BB_ [42]. Strikingly, these cells are contractile (**Figure 5**) despite presenting a small percentage of polymerized actin fibers (6%; **Figure S3**). Nevertheless, it is known that the total amount of actin that undergoes polymerization after induction of contraction is relatively small [43]. Further research is needed to study the molecular processes that regulate the assembly of actin filaments in smooth muscle tissues and the nature of the actin filaments network that are formed during contractile activation [43].

Contractile and synthetic SMCs, which represent the two ends of a spectrum of SMCs with intermediate phenotypes, clearly show different morphologies [22]. The SMPCs derived in this work seem to have the contractile phenotype, as they are spindle-shaped [44], express proteins involved in SMC contraction including α-SMA, SM-MHC, calponin and SMα-22 [22], [24], and contract when exposed to carbachol and vasoactive peptides being this effect reversed by the presence of the respective antagonists.

We show that SMPCs are able to contract to a plethora of vasoactive agents including carbachol, angiotensin II, histamine, thromboxane-mimetic U46619, endothelin-1, and bradykinin, as hVSMCs. Most of these agonists act through different receptors coupled to G-proteins activating membrane-bound phospholipase C, which leads to the formation of inositol-1,4,5-triphosphate (IP_3_) and diacylglycerol (DAG) [26]. Through different mechanisms, both molecules induce the accumulation of Ca^2+^ in the cell cytoplasm [26]. The increase of intracellular Ca^2+^ activates the cell's contraction machinery. Our results agree with previous ones showing that hESC-derived SMCs respond to bradykinin, histamine and carbachol by increasing at different levels the intracellular concentration of Ca^2+^ [14]. The accumulation of intracellular Ca^2+^ in hESC-derived SMCs exposed to carbachol is minimal, indicating that the contraction of the cell may involve the activation of other intracellular players (e.g. protein kinase C) than Ca^2+^, as described in other studies [45].

Our results show that hESC-derived SMPC contraction is mediated by the activation of CaM/MLCK- and Rho/Rho kinase-dependent pathways [7], [26]. A similar mechanism has been reported recently for SMLCs obtained from human adipose tissue-derived mesenchymal stem cells [7]. The results also indicate that the absence of fully organized protein filaments did not prevent the contraction of SMLCs. This is in line with results reported previously by us [12] and by others [13] despite the absence of maturity and organization in the contractile machinery of the cell.

SMCs have key biological functions in terms of contraction and secretion of soluble signaling molecules [46]. In the present study we report for the first time the secretion profile of hES-derived SMPCs. CD34^+^ cells that differentiate in medium supplemented with PDGF_BB_ have similar secretome profile as hVSMCs. They express high levels (>100 pg/mL) of IL-6 and IL-8, moderate levels (between 100 and 10 pg/mL) of IFN-γ, and small levels of (between 10 and 1 pg/mL) of IL-7, G-CSF, MCP-1 and TNF-α. IL-6 is a cytokine with potent inflammatory properties and metabolic effects in SMCs. It has been shown that IL-6 secretion is inversely correlated to glucose consumption [47]. IL-8 is a cytokine that induces the proliferation and chemotaxis of smooth muscle cells and increases the activity of mitogen-activated protein kinase (MAPK) [48]. IFN-γ acts on vascular smooth muscle cells to induce cellular proliferation [49].

Although several studies have reported the differentiation of different stem cells into SMCs, very few evaluated the impact of the 3D environment at the geno- and phenotype of the differentiated cells [5]. Gene studies have demonstrated that human aortic SMCs encapsulated in a 3D collagen matrix have significantly less focal adhesions, lower proliferation, and lower α-SMA expression than cells in 2D [50]. However, to our knowledge, no study has compared the gene expression of SMLCs from different origins with fully differentiated SMCs when cultured in 2D or 3D systems. Our results show that hESC-derived SMPCs encapsulated for 3 days in fibrin gels express similar gene levels of α-SMA, SM-MHC, and SMα22 as encapsulated hVSMCs. In addition, considering an array of 84 genes related to cell-cell and cell-matrix interactions, 3D-cultured-SMPCs had a more hVSMC-similar gene expression profile than 2D-cultured SMPCs. This shows that 3D scaffolds may induce further the differentiation of SMPCs into SMCs. Several factors might account for the differences found between 2D and 3D culture systems including (i) ECM stiffness and (ii) ECM 3D environment. It has been demonstrated that the stiffness of the ECM has a high impact in the cytoskeletal and focal adhesion assembly of SMCs [51]. Furthermore, SMCs cultured within 3D polyethylene glycol-fibrinogen [52] or collagen [50] gels had less proliferation, stress fibers and focal adhesion than on 2D culture systems. Future studies should evaluate the effect of both factors in the modulation of geno- and phenotype of the differentiated cells over the time and study the underlining mechanism. In this study we assessed the effect of the 3D matrix after 3 days of cell encapsulation. During this time, previous studies have shown that SMCs are able to migrate and remodel the ECM [53]; however, it would be interesting to extend these studies in time.

Although very recent studies have identified and characterized hESC-derived populations with SMC potential [13], [14], identified bioactive agents involved in their SMC differentiation, and evaluated their functionality, our study advance these results in several ways. First, it provides a detailed analysis of the phenotype, secretome and function (unraveling the mechanism of cell contraction) of hESC-derived SMCs at levels not documented before. In contrast to the previous studies that have used monolayer-based differentiation protocols, our methodology uses an EB-differentiation step to isolate progenitor cells, which might have advantages for the scale-up of the process through the use of bioreactors. Second, our study identifies a methodology to induce the organization of the contractile protein filaments. Third, it demonstrates the importance of a 3D environment to modulate the activity of hESC-derived SMLCs.

Recent studies reported the derivation of pluripotent stem cells from human somatic cells by retroviral transduction [54]. These reprogrammed cells share many features with hESCs in terms of morphology, gene expression and differentiation potential, and open a new avenue for the potential derivation of autologous cells for regenerative medicine and drug screening. Indeed, the differentiation of iPSCs into SMCs has been recently reported [55]. It remains to be determined whether the methodology described in this work can be applied on iPSCs and this is an issue that will be evaluated in future work.

## Supporting Information

Figure S1 **SMC proteins are expressed on hESC-derived SMPCs.** CD34^+^, CD34^+^KDR^−^ and CD34^−^ cells differentiated under different media conditions express α-SMA, calponin and SM-MHC, as evaluated by immunofluorescence. hVSMCs were used as a positive control and HUVECs as a negative control for the SMC markers. In all figures, bar corresponds to 50 µm.
(TIFF)

Figure S2 **Isotype controls for SMPC immunostainning.** Stained the isotype controls IgG_2A_ and IgG_1_ for the SMC markers: α-SMA, SM-MHC and Calponin. Cell nuclei were stained with 4′, 6′-diamidino-2-phenylindole (DAPI). Cells were labeled with mouse anti–human IgG_2A_ and IgG_1_ antibodies. Bar corresponds to 50 µm.
(TIFF)

Figure S3 **Organization and expression of contractile proteins.** (**A**) Quantification of organized α-SMA, SM-MHC and calponin filaments. (B) Expression of α-SMA in differentiated CD34^+^, CD34^+^KDR^−^ and CD34^−^ cells. Cells were differentiated for 3 passages (approximately 18 days after cell seeding). hVSMCs and HUVECs were used as positive and negative controls, respectively. In all graphs, the percentages of positive cells were calculated based in the isotype controls (gray plot) and are shown in each histogram plot.
(TIFF)

Figure S4 **Secretomic profile of hESC-derived cells.** 17 cytokines were measured simultaneously in the medium collected from CD34^+^KDR^−^PDGF_BB_ and CD34^+^KDR^−^EGM-2 cells. A standard range of 0.2 to 3,200 pg/mL was used. Samples and controls were run in triplicate, standards and blanks in duplicate.
(TIFF)

Figure S5 **Contractility of hESC-derived cells.** Cells were loaded with FURA-2/AM and their response to carbachol (10^−5^ M) was monitored by fluorescence. The response profile was compared to the one observed for hVSMCs and HUVECs, as positive and negative controls, respectively.
(TIFF)

Figure S6 **Contractility of hESC-derived cells.** Cells were loaded with FURA-2/AM and their response to vasoactive agonists (bradykinin (10^−7^ M), angiotensin II (10^−5^ M) and histamine (100 µM) and depolarization agents (KCl; 50 mM) was monitored by fluorescence. The response profile was compared to the one observed for hVSMCs and HUVECs, as positive and negative controls, respectively.
(TIFF)

Figure S7 **Integrin gene expression.** Gene expression on CD34^+^PDGF_BB_ and CD34^+^RA cells was normalized by gene expression on hVSMCs, both cultured in 3D or 2D systems. Gene expression was obtained from the RT^2^ Profiler™ PCR array.
(TIFF)

Figure S8 **Expression and organization of SMC proteins on CD34^−^PDGF_BB_ cells treated with vasoactive agents for 3 days.** A) Quantification by immunocytochemistry analysis. Results are Mean ± SEM (*n* = 8). B) Expression of a-SMA and calponin in CD34^−^PDGF_BB_ cells treated with End-1 (10 nM) for 3 days. Bar corresponds to 50 µm.
(TIFF)

Figure S9 **Gene expression in CD34^+^PDGF_BB_ and CD34^+^RA cells after treatment with vasoactive agents for 3 days.** Gene expression in CD34^+^PDGF_BB_ and CD34^+^RA cells was normalized by gene expression in hVSMCs. Results are Mean ± SEM (*n* = 4).
(TIFF)

Figure S10 **Expression and organization of troponin T (TrpnT) and vascular endothelial-cadherin (VECad) on CD34^+^RA (A) and CD34^+^PDGF_BB_ (B) cells treated with End-1 for 3 days.** Human cardiac tissue (for TrpnT) and HUVECs (for VeCad) were used as positive controls. Bar corresponds to 50 µm.
(TIFF)

Table S1 **Primers used for Real Time PCR.** PCR conditions: initial denaturation step at 94°C for 5 min; 40 cycles of denaturation at 94°C for 30 sec, annealing at 60°C for 33 sec and extension at 72°C for 30 sec. At the end was performed a final 7 minutes extension at 72°C. After amplification, the melting curve profile or agarose gel electrophoresis was used to determine the specificity of PCR products.
(TIFF)

Materials and Methods S1 (DOC)
