# Supplementary figures and images for: Towards the Maturation and Characterization of Smooth Muscle Cells Derived from Human Embryonic Stem Cells
Source: PLoS One. 2011 Mar 10;6(3):e17771. doi: 10.1371/journal.pone.0017771 (PMC3053392; doi:10.1371/journal.pone.0017771)

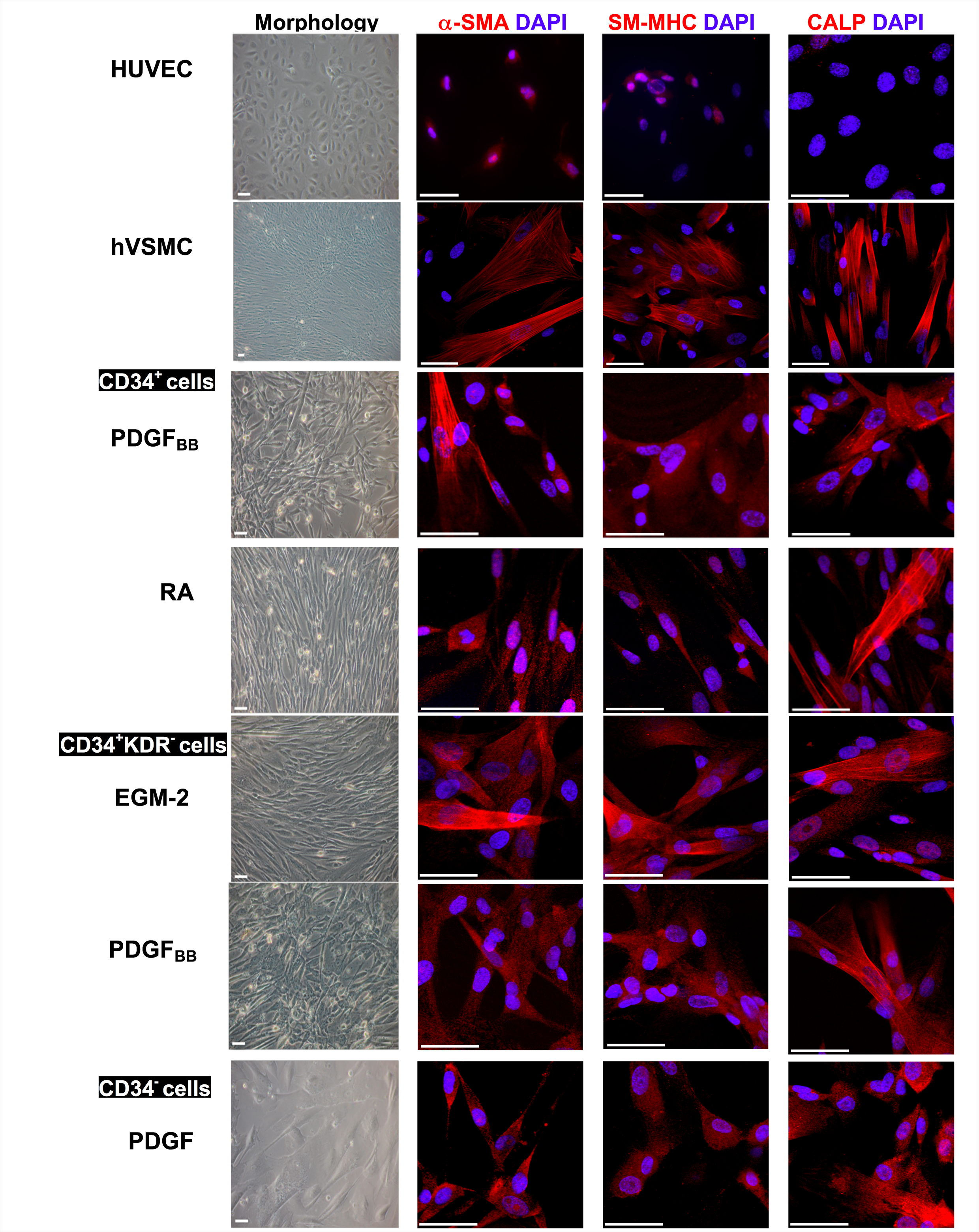

Supplement: Figure S1 — SMC proteins are expressed on hESC-derived SMPCs. CD34+, CD34+KDR− and CD34− cells differentiated under different media conditions express α-SMA, calponin and SM-MHC, as evaluated by immunofluorescence. hVSMCs were used as a positive control and HUVECs as a negative control for the SMC markers. In all figures, bar corresponds to 50 µm. (TIFF) [file pone.0017771.s001.tif]

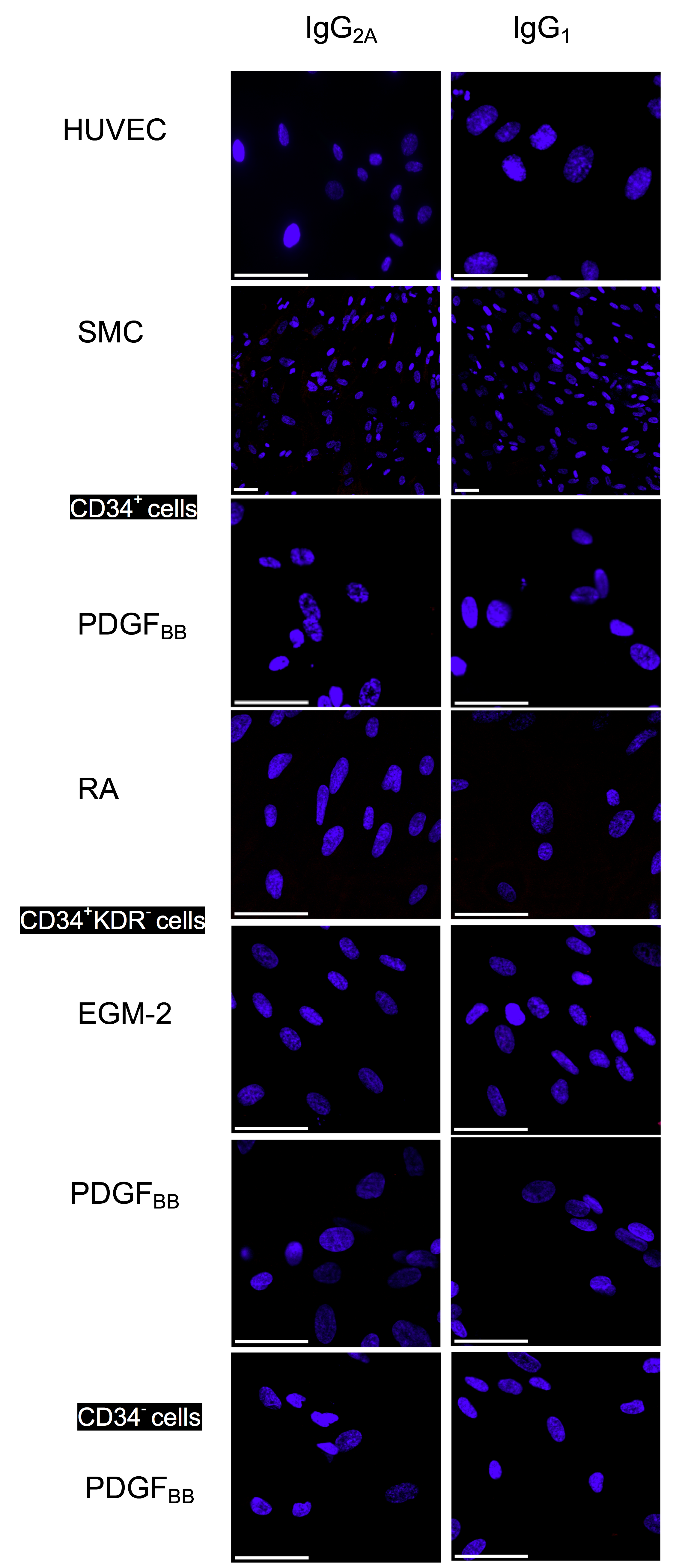

Supplement: Figure S2 — Isotype controls for SMPC immunostainning. Stained the isotype controls IgG2A and IgG1 for the SMC markers: α-SMA, SM-MHC and Calponin. Cell nuclei were stained with 4′, 6′-diamidino-2-phenylindole (DAPI). Cells were labeled with mouse anti–human IgG2A and IgG1 antibodies. Bar corresponds to 50 µm. (TIFF) [file pone.0017771.s002.tif]

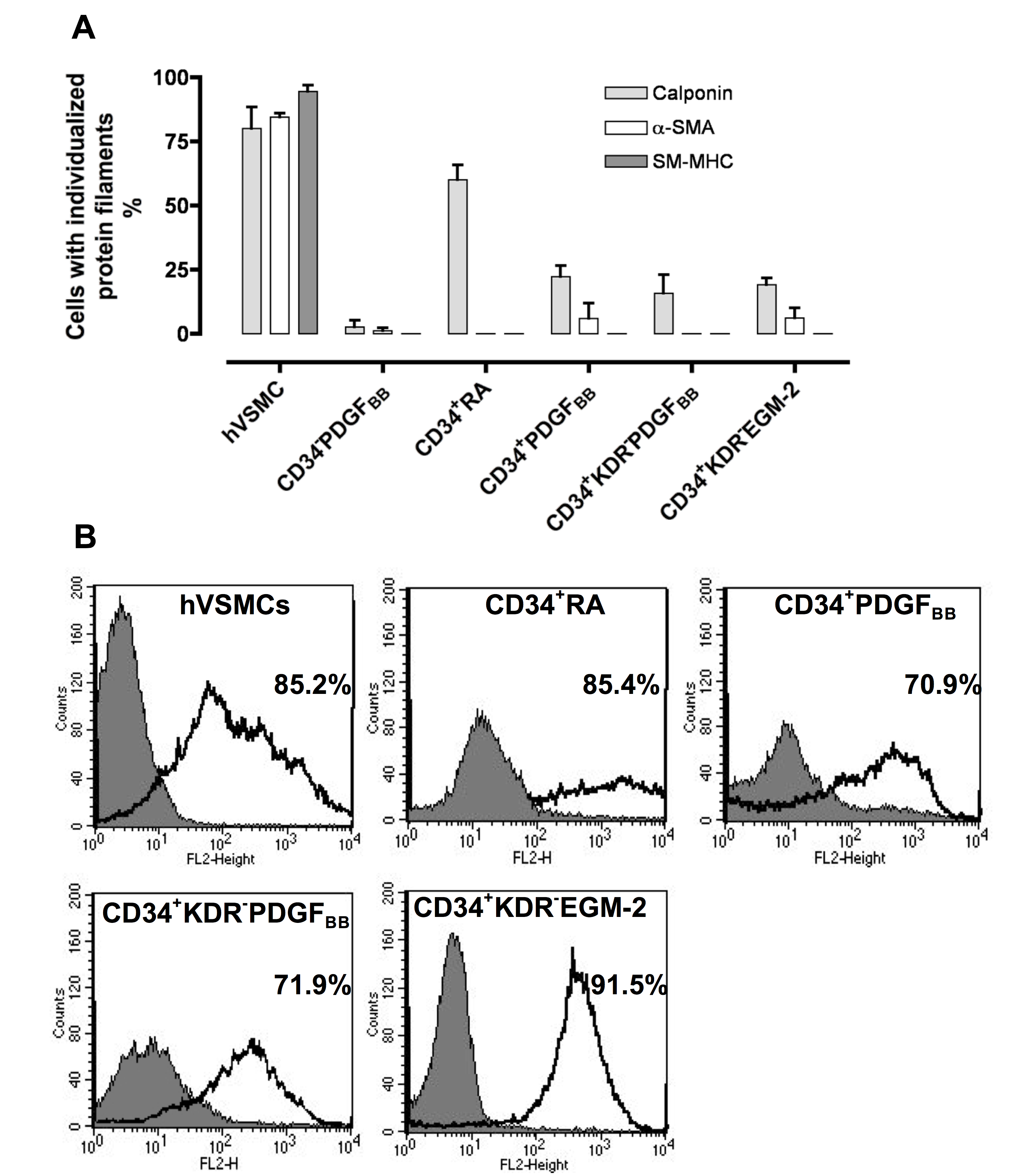

Supplement: Figure S3 — Organization and expression of contractile proteins. (A) Quantification of organized α-SMA, SM-MHC and calponin filaments. (B) Expression of α-SMA in differentiated CD34+, CD34+KDR− and CD34− cells. Cells were differentiated for 3 passages (approximately 18 days after cell seeding). hVSMCs and HUVECs were used as positive and negative controls, respectively. In all graphs, the percentages of positive cells were calculated based in the isotype controls (gray plot) and are shown in each histogram plot. (TIFF) [file pone.0017771.s003.tif]

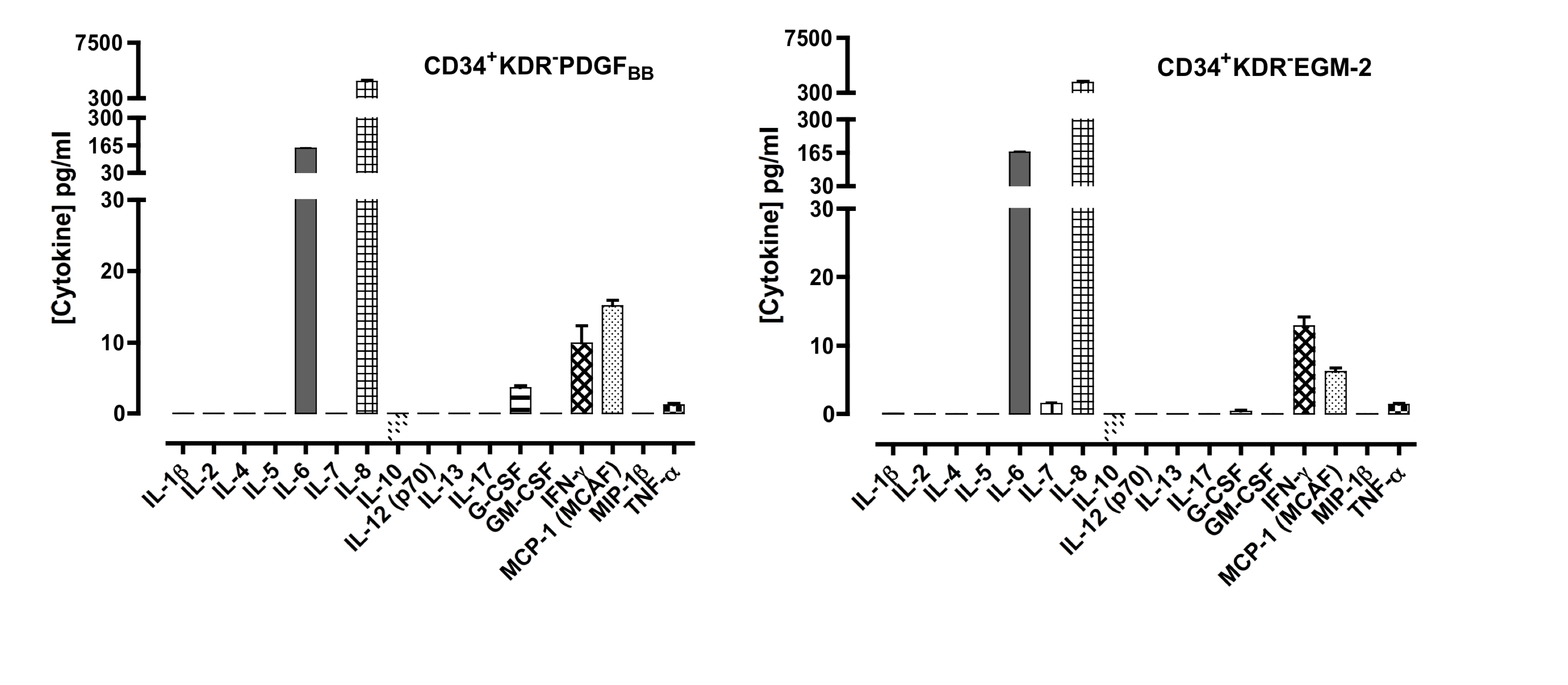

Supplement: Figure S4 — Secretomic profile of hESC-derived cells. 17 cytokines were measured simultaneously in the medium collected from CD34+KDR−PDGFBB and CD34+KDR−EGM-2 cells. A standard range of 0.2 to 3,200 pg/mL was used. Samples and controls were run in triplicate, standards and blanks in duplicate. (TIFF) [file pone.0017771.s004.tif]

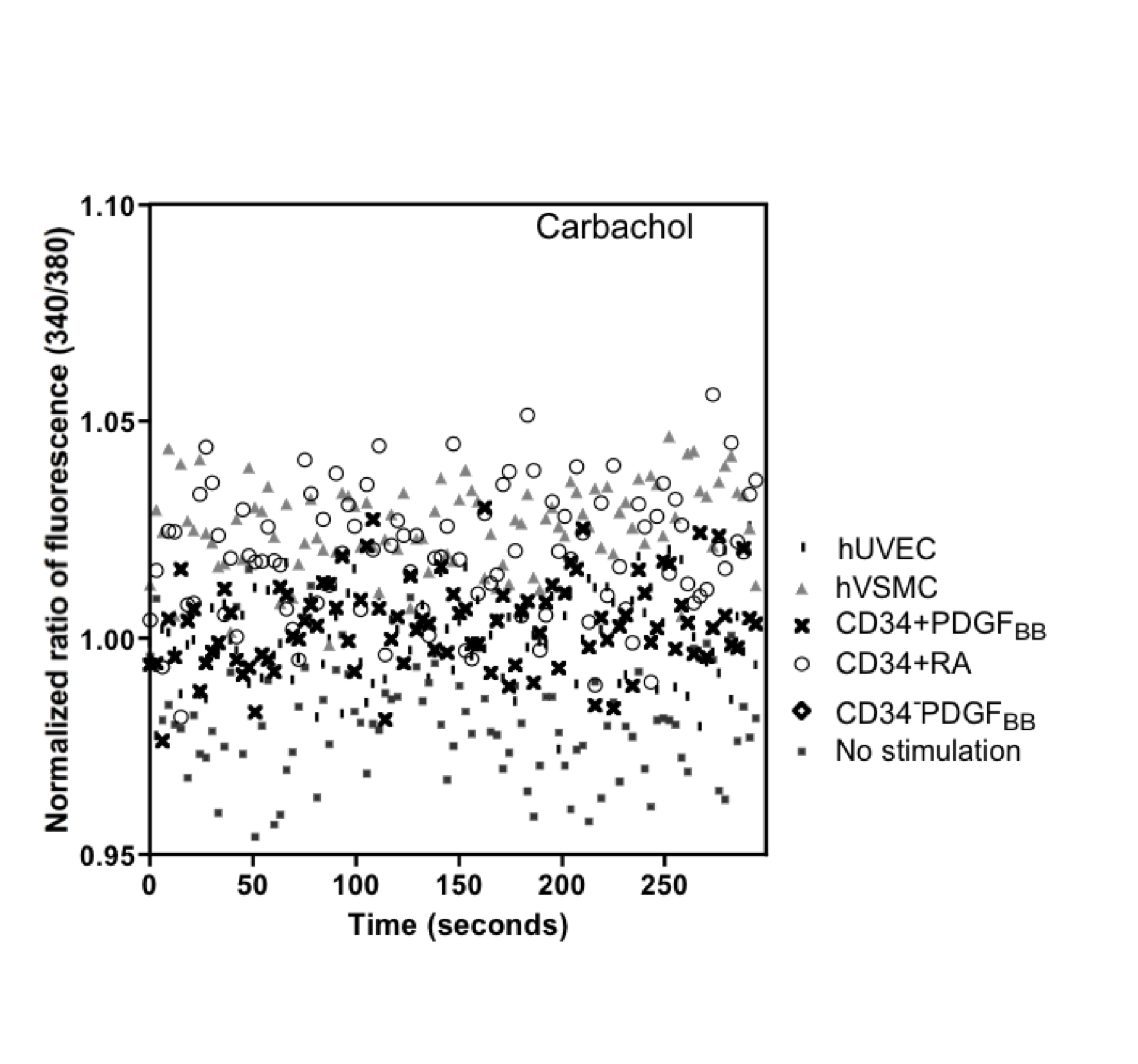

Supplement: Figure S5 — Contractility of hESC-derived cells. Cells were loaded with FURA-2/AM and their response to carbachol (10−5 M) was monitored by fluorescence. The response profile was compared to the one observed for hVSMCs and HUVECs, as positive and negative controls, respectively. (TIFF) [file pone.0017771.s005.tif]

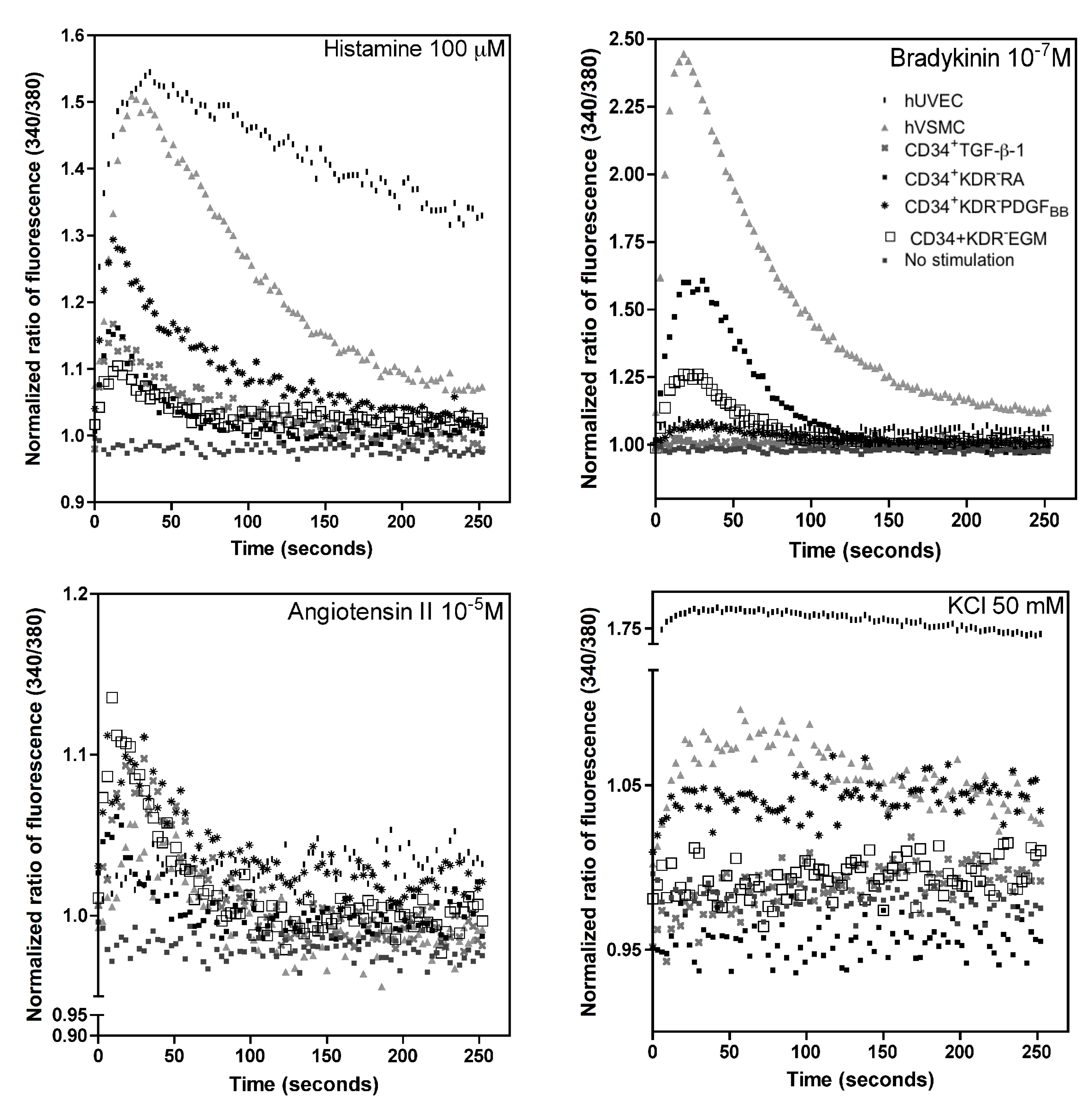

Supplement: Figure S6 — Contractility of hESC-derived cells. Cells were loaded with FURA-2/AM and their response to vasoactive agonists (bradykinin (10−7 M), angiotensin II (10−5 M) and histamine (100 µM) and depolarization agents (KCl; 50 mM) was monitored by fluorescence. The response profile was compared to the one observed for hVSMCs and HUVECs, as positive and negative controls, respectively. (TIFF) [file pone.0017771.s006.tif]

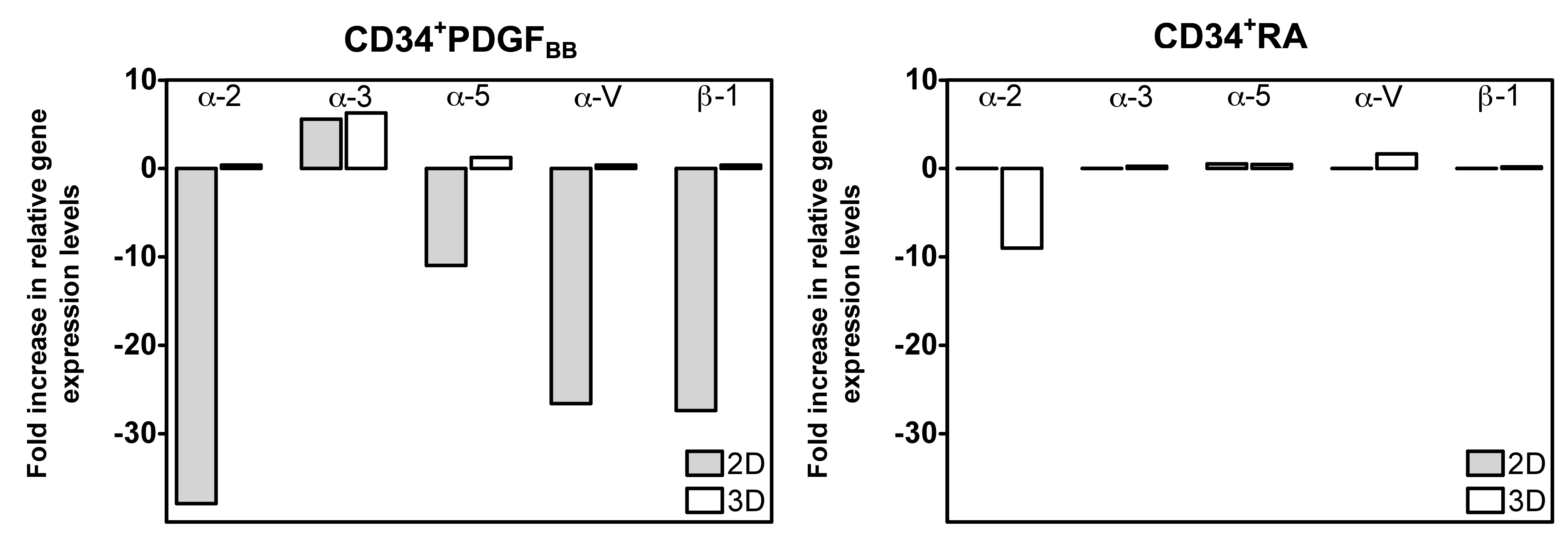

Supplement: Figure S7 — Integrin gene expression. Gene expression on CD34+PDGFBB and CD34+RA cells was normalized by gene expression on hVSMCs, both cultured in 3D or 2D systems. Gene expression was obtained from the RT2 Profiler™ PCR array. (TIFF) [file pone.0017771.s007.tif]

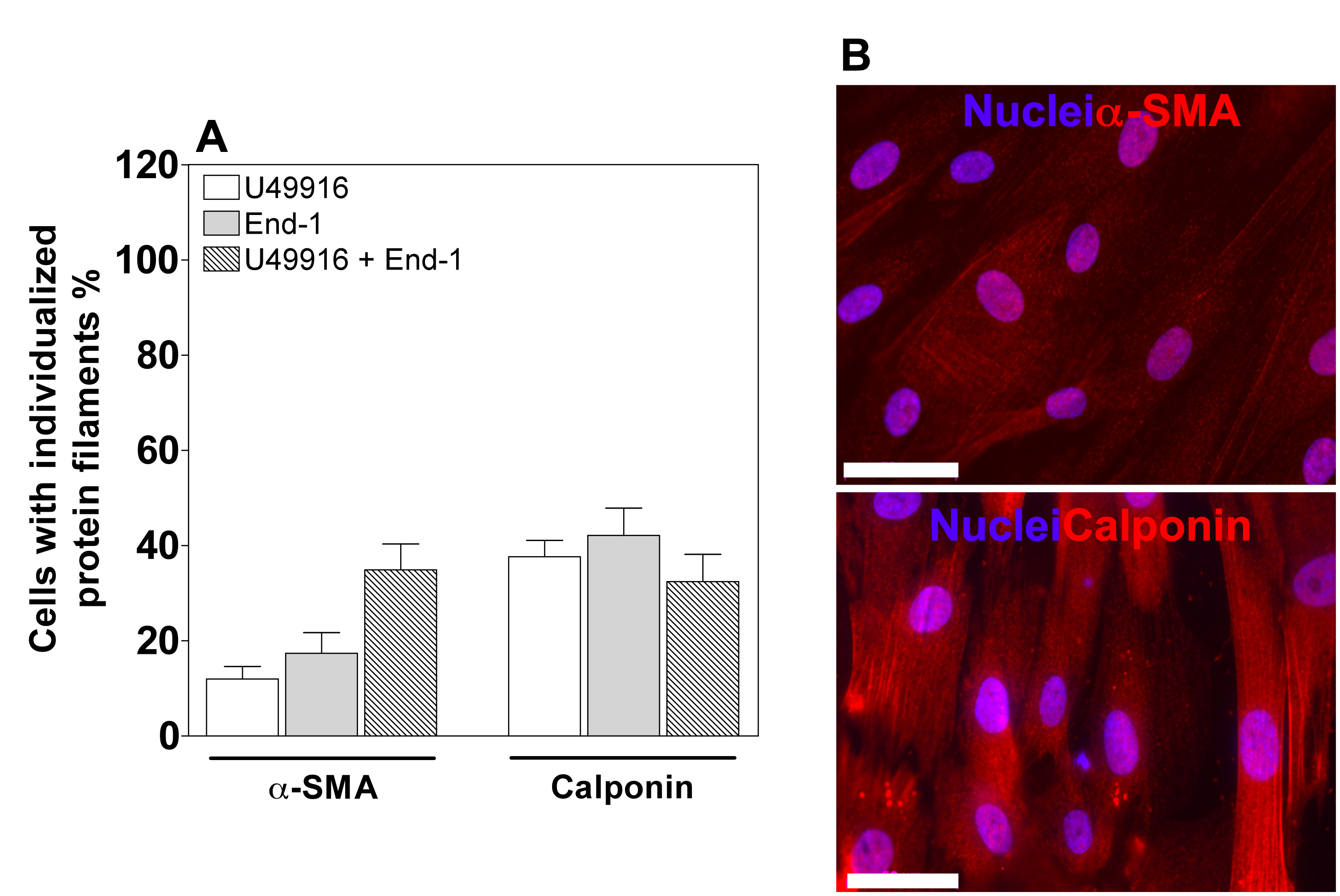

Supplement: Figure S8 — Expression and organization of SMC proteins on CD34−PDGFBB cells treated with vasoactive agents for 3 days. A) Quantification by immunocytochemistry analysis. Results are Mean ± SEM (n = 8). B) Expression of a-SMA and calponin in CD34−PDGFBB cells treated with End-1 (10 nM) for 3 days. Bar corresponds to 50 µm. (TIFF) [file pone.0017771.s008.tif]

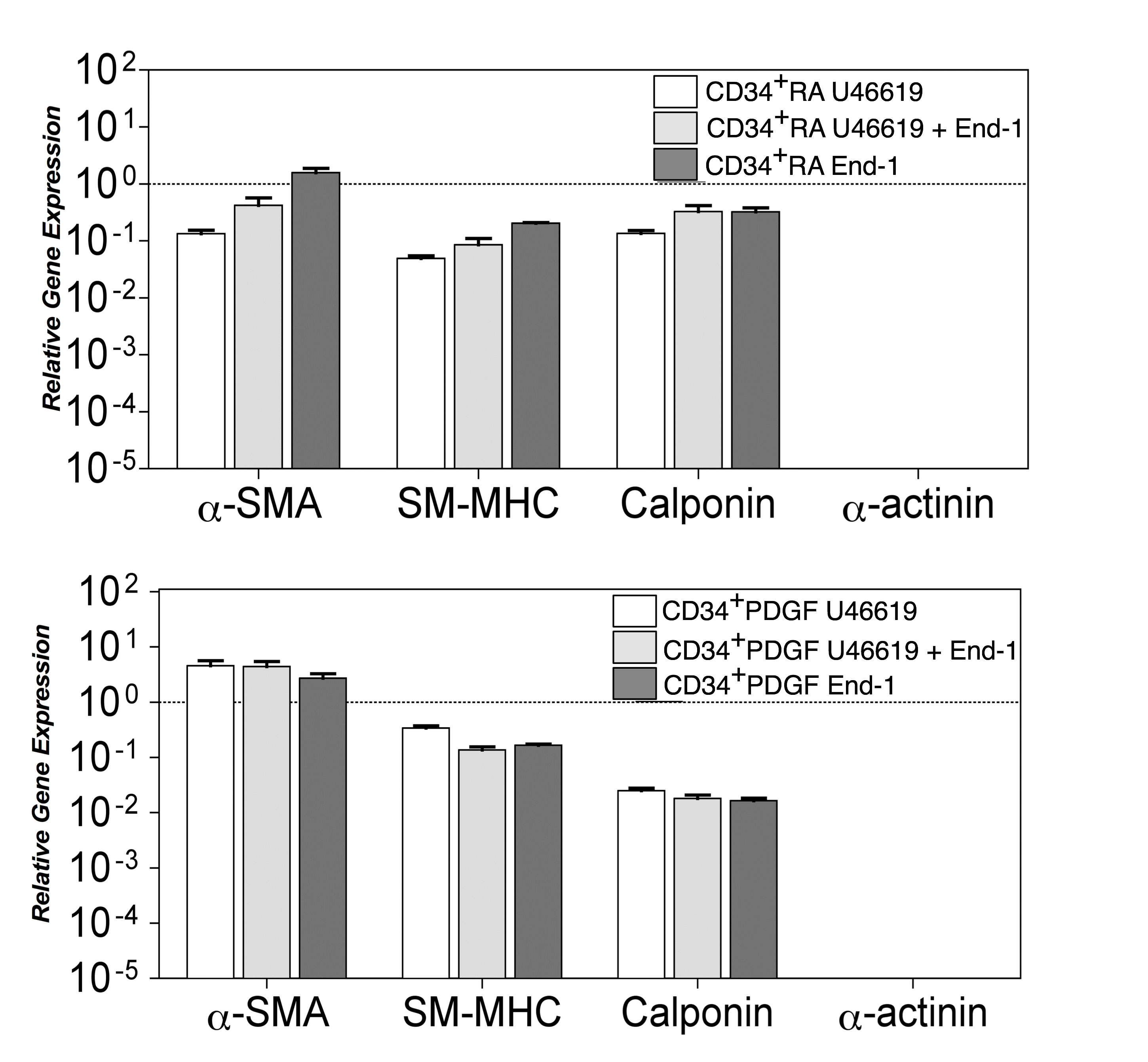

Supplement: Figure S9 — Gene expression in CD34+PDGFBB and CD34+RA cells after treatment with vasoactive agents for 3 days. Gene expression in CD34+PDGFBB and CD34+RA cells was normalized by gene expression in hVSMCs. Results are Mean ± SEM (n = 4). (TIFF) [file pone.0017771.s009.tif]

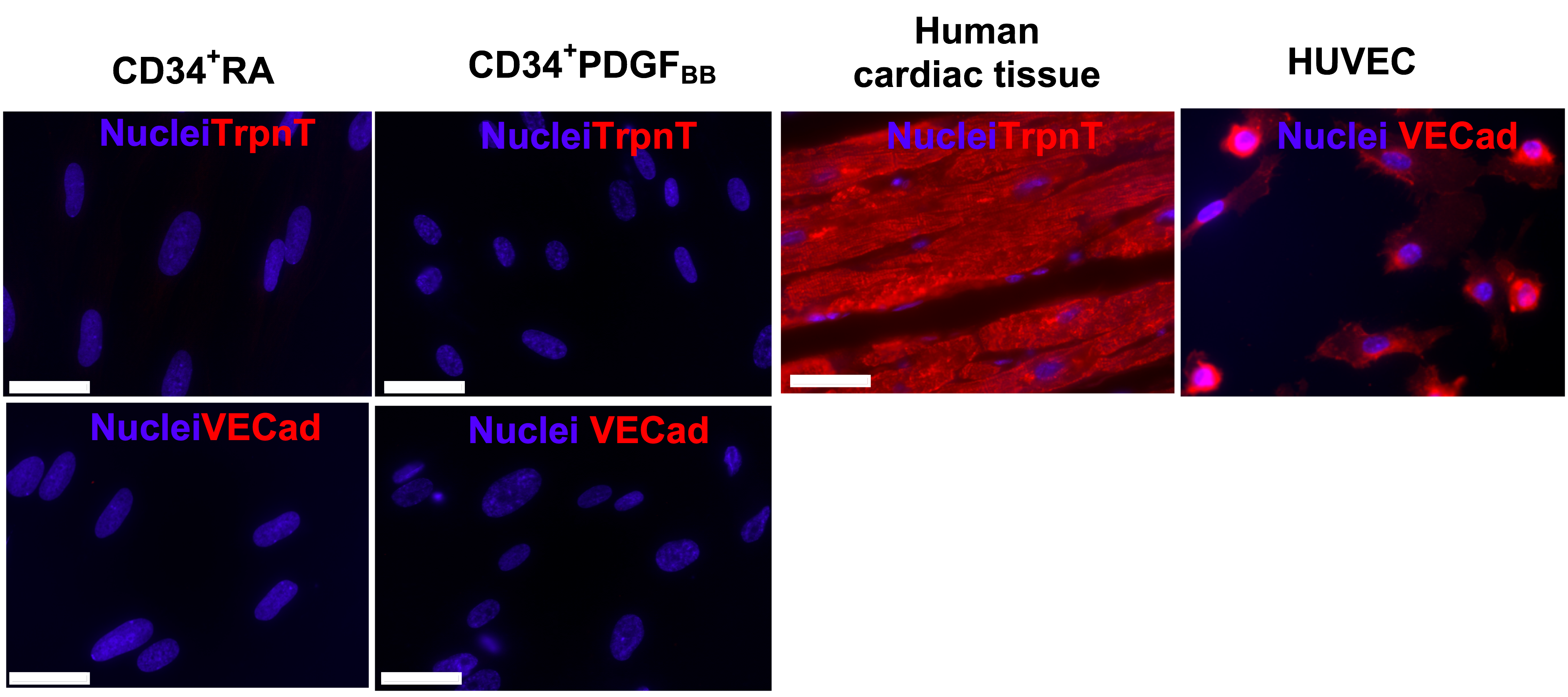

Supplement: Figure S10 — Expression and organization of troponin T (TrpnT) and vascular endothelial-cadherin (VECad) on CD34+RA (A) and CD34+PDGFBB (B) cells treated with End-1 for 3 days. Human cardiac tissue (for TrpnT) and HUVECs (for VeCad) were used as positive controls. Bar corresponds to 50 µm. (TIFF) [file pone.0017771.s010.tif]

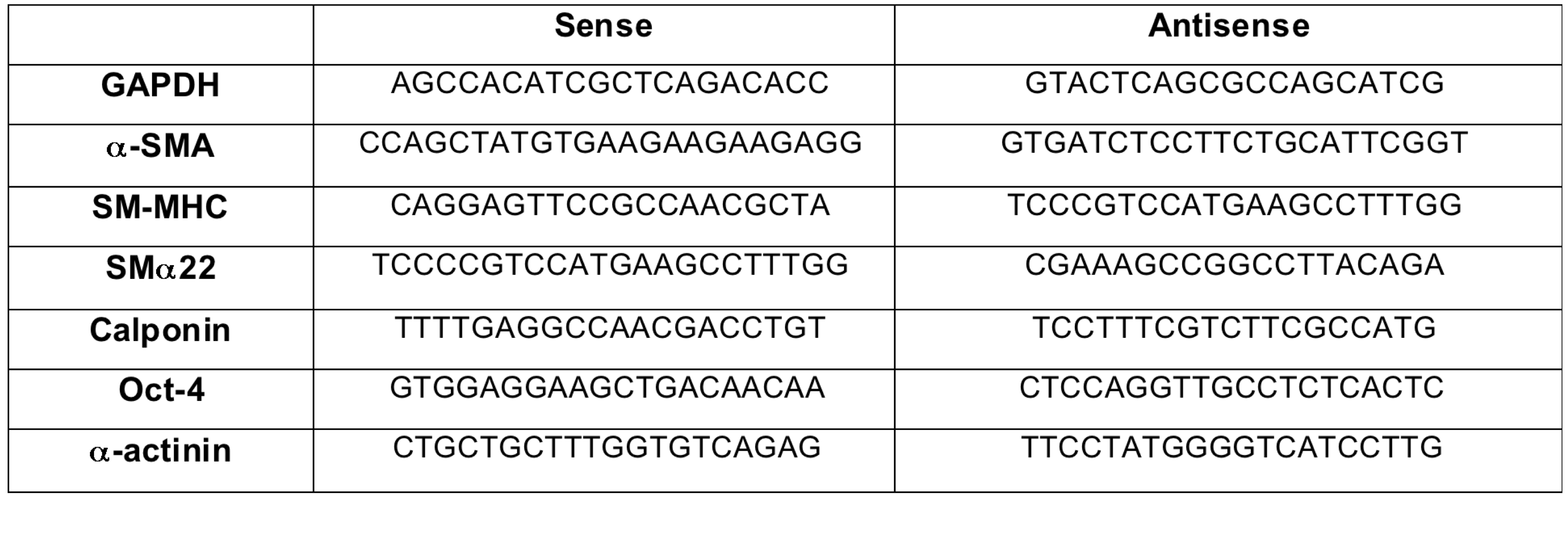

Supplement: Table S1 — Primers used for Real Time PCR. PCR conditions: initial denaturation step at 94°C for 5 min; 40 cycles of denaturation at 94°C for 30 sec, annealing at 60°C for 33 sec and extension at 72°C for 30 sec. At the end was performed a final 7 minutes extension at 72°C. After amplification, the melting curve profile or agarose gel electrophoresis was used to determine the specificity of PCR products. (TIFF) [file pone.0017771.s011.tif]
