# Supplementary material for: Towards the Maturation and Characterization of Smooth Muscle Cells Derived from Human Embryonic Stem Cells
Source: PLoS One. 2011 Mar 10;6(3):e17771. doi: 10.1371/journal.pone.0017771 (PMC3053392; doi:10.1371/journal.pone.0017771)
Supplement: Materials and Methods S1 — (DOC) [file pone.0017771.s012.doc]

**Materials and Methods S1:**

**Towards the maturation and characterization of smooth muscle cells derived from human embryonic stem cells**

Helena Vazão^1,2^, Ricardo Pires das Neves^1,2^, Mário Grãos^2^, Lino Ferreira^1,2^*.

^1^ CNC - Center of Neurosciences and Cell Biology, University of Coimbra, Portugal;

^2^ Biocant - Center of Innovation in Biotechnology, Cantanhede, Portugal.

**Expanded Materials and Methods**

**Flow cytometry analysis**

Cells were tripsinized, aliquoted (1.25-2.5 × 10^5^ cells per condition) and fixed for 10 minutes in cold 4% (v/v, in PBS) paraformaldehyde (Electron Microscopy Sciences). Afterwards, the cells were washed in PBS, centrifuged at 1200 g and ressuspended in permeabilization buffer (PBS containing 0.5% Triton X-100) for 7 minutes. Cells were incubated in permeabilization buffer containing human smooth muscle α-actin monoclonal antibody (R&D) or isotype control IgG2a antibody (R&D). Cells were characterized by using a FACS Calibur (BD) and the data analysed by Cell Quest software. Twenty thousand events were collected in each run.

**Intracellular Ca^2+^ variation measurements**

SMCs or hESC-derived SMPCs were loaded with Fura-2 calcium fluorescent indicator by incubation with 10 μM of the membrane permeable acetoxymethyl (AM) derivative FURA-2/AM (1 mM in DMSO, Molecular Probes) and 0.06% (w/v) Pluronic F-127 (Sigma), using basal medium (M199, Sigma) as vehicle (35 μl/well, not supplemented with serum or antibiotics), for 1 h at 37ºC in 5% CO_2_ and 90% humidity. The medium was then replaced by the respective basal medium and cells were incubated in the same conditions for 30 min to allow hydrolysis of the acetoxymethyl (AM) esters by cellular esterases, resulting in intracellular capture of the membrane impermeant Fura-2. Cells were then washed twice with 100 μL sodium salt solution (140 mM NaCl, 5 mM KCl, 1 mM CaCl_2_, 1 mM MgCl_2_, 10 mM Glucose, 10 mM HEPES-Na^+^ pH 7.4). The buffer was then replaced again (100 μl/well) immediately prior to incubating or not with test compounds.

Cells located in wells on a plate row were incubated at 25ºC (inside the microplate reader, during basal reading). Cells were then stimulated with 100 μM Histamine (Sigma) [1,2], 10^-7^ M Bradykinin (Calbiochem) [3], 10^-5^ M Angiotensin II (Calbiochem) [3] or 50 mM KCl (Merck) [1,2] by adding 1 μl of a stock solution.

Fluorescence was measured at emission 510 nm using two alternating excitation wavelengths (340nm and 380nm) [4] using a microplate fluorescence reader (Spectramax Gemini EM, Molecular Devices, with SoftMax® Pro software). The microplate reader was set to “top-read kinetics”; PMT was “high”; temperature was 25ºC; each fluorescence time point was an average of 18 reads; and during basal/inhibitors incubation periods and KCl/Histamine stimulation, each well was read every 3 sec. Fluorescence intensity values (in relative fluorescence units – RFU) measured at 340 nm and 380 nm were taken from the stabilized signal obtained at basal conditions and after incubation or not with the inhibitors. Following stimulation with Histamine/Bradykinin/Angiotensin II/KCl, the fluorescence values were taken from the time point at the peak of the response. Typically, each experiment consisted of three or four wells plus three or four wells containing cells incubated with inhibitors and all cells were stimulated and read simultaneously. The dose-response curves for the effect of stimuli on the intracellular Ca^2+^ variation were determined using the software GraphPad Prism^TM^.

**Cytokine measurements**

Cell culture supernatants were assayed for cytokines using a Bio-plex human 17-plex panel immunoassay kit (Bio-Rad), according to manufacturer’s instructions. The 17-Plex panel consisted of the following analytes: interleukin-1 (IL-1β), IL-2, IL-4, IL-5, IL-6, IL-7, IL-8; IL-10, IL-12(p70), IL-13, IL-17, granulocyte colony-stimulating factor (G-CSF), granulocyte/macrophage colony-stimulating factor (GM-CSF), interferon (IFN-γ), monocyte chemotactic protein (MCP-1 (MCAF)), macrophage inﬂammatory protein (MIP-1b), and tumor necrosis factor (TNF-α). A standard range of 0.2 to 3,200 pg/mL was used. Supernatants media samples were collected, centrifuged and frozen. Samples and controls were run in triplicate, standards and blanks in duplicate.

**References:**

1. Agasse F, Bernardino L, Kristiansen H, Christiansen SH, Ferreira R, et al. (2008) Neuropeptide Y promotes neurogenesis in murine subventricular zone. Stem Cells 26: 1636-1645.

2. Bernardino L, Agasse F, Silva B, Ferreira R, Grade S, et al. (2008) Tumor necrosis factor-alpha modulates survival, proliferation, and neuronal differentiation in neonatal subventricular zone cell cultures. Stem Cells 26: 2361-2371.

3. Drab M, Haller H, Bychkov R, Erdmann B, Lindschau C, et al. (1997) From totipotent embryonic stem cells to spontaneously contracting smooth muscle cells: a retinoic acid and db-cAMP in vitro differentiation model. FASEB J 11: 905-915.

4. Grynkiewicz G, Poenie M, Tsien RY (1985) A new generation of Ca2+ indicators with greatly improved fluorescence properties. J Biol Chem 260: 3440-3450.
